# Gene expression profiling reveals effects of *Cimicifuga racemosa *(L.) NUTT. (black cohosh) on the estrogen receptor positive human breast cancer cell line MCF-7

**DOI:** 10.1186/1471-2210-7-11

**Published:** 2007-09-20

**Authors:** Friedemann Gaube, Stefan Wolfl, Larissa Pusch, Torsten C Kroll, Matthias Hamburger

**Affiliations:** 1 Institute of Pharmacy, Department of Pharmaceutical Biology, University of Jena, Semmelweisstr. 10, 07743 Jena, Germany; 2 Clinic of Internal Medicine II, University of Jena, Erlanger Allee 101, 07747 Jena, Germany; 3 Institute of Pharmacy and Molecular Biotechnology, University of Heidelberg, Im Neuenheimerfeld 364, 69120 Heidelberg, Germany; 4 Department of Pharmaceutical Sciences, Institute of Pharmaceutical Biology, University of Basel, Klingelbergstr. 50, CH-4053 Basel, Switzerland

## Abstract

**Background:**

Extracts from the rhizome of *Cimicifuga racemosa *(black cohosh) are increasingly popular as herbal alternative to hormone replacement therapy (HRT) for the alleviation of postmenopausal disorders. However, the molecular mode of action and the active principles are presently not clear. Previously published data have been largely contradictory. We, therefore, investigated the effects of a lipophilic black cohosh rhizome extract and cycloartane-type triterpenoids on the estrogen receptor positive human breast cancer cell line MCF-7.

**Results:**

Both extract and purified compounds clearly inhibited cellular proliferation. Gene expression profiling with the extract allowed us to identify 431 regulated genes with high significance. The extract induced expression pattern differed from those of 17β-estradiol or the estrogen receptor antagonist tamoxifen. We observed a significant enrichment of genes in an anti-proliferative and apoptosis-sensitizing manner, as well as an increase of mRNAs coding for gene products involved in several stress response pathways. These functional groups were highly overrepresented among all regulated genes. Also several transcripts coding for oxidoreductases were induced, as for example the cytochrome P450 family members 1A1 and 1B1. In addition, some transcripts associated with antitumor but also tumor-promoting activity were regulated. Real-Time RT-PCR analysis of 13 selected genes was conducted after treatment with purified compounds – the cycloartane-type triterpene glycoside actein and triterpene aglycons – showing similar expression levels compared to the extract.

**Conclusion:**

No estrogenic but antiproliferative and proapoptotic gene expression was shown for black cohosh in MCF-7 cells at the transcriptional level. The effects may be results of the activation of different pathways. The cycloartane glycosides and – for the first time – their aglycons could be identified as an active principle in black cohosh.

## Background

*Cimicifuga racemosa *(L.) NUTT. (syn. *Actaea racemosa*; black cohosh, family Ranunculaceae) is a North American perennial herb which has been traditionally used by Native Americans for the treatment of rheumatism, dyspepsia, epilepsy, kidney ailments, dysmenorrhoea and the relief of pain during menses and childbirth [1,2]. Since the 1950s, phytopharmaceuticals containing black cohosh extracts from the rhizome are used for the alleviation of menopausal disorders [3]. Since the Women's Health Initiative (WHI) described adverse effects of hormone replacement therapy (HRT) such as increased risk of breast cancer and cardiovascular diseases [4], the popularity of herbal alternatives such as black cohosh has considerably increased.

**Figure 1:**
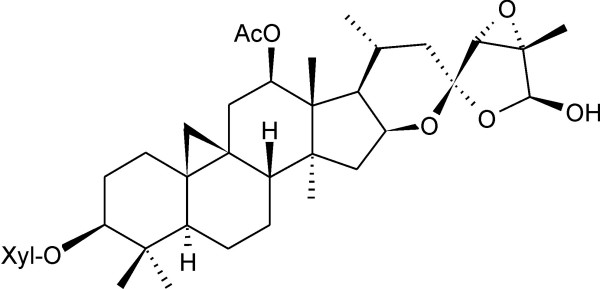
Chemical structure of Actein, the major cycloartane-type triterpene glycoside of black cohosh.

The phytochemicals in black cohosh rhizomes have been well studied, and numerous cycloartane-type triterpene glycosides (e.g. actein Figure 1), aromatic acids (e.g. caffeic and ferulic acid), cinnamic acid esters (e.g. fukinolic acid, cimicifugic acids) and various minor compounds have been reported [1,2,5]. The pharmacology of rhizome extracts has been investigated, whereby most investigations addressed the effects on the hormonal (estrogenic) system. An estrogen-like activity was reported initially [6-8], but these effects could not be confirmed in later studies [8-11]. Subsequently, antiestrogenic properties were proposed on the basis of *in vitro *experiments [8,12,13]. Because a black cohosh extract influenced bone, endometrium and hypothalamus, but not uterus in a estrogen-like manner, it was postulated to contain compounds that act as selective estrogen receptor modulators (SERMs) and, therefore, to be different from common phytoestrogens – a presumption that is still a matter of dispute [14-17].

In binding studies black cohosh extracts showed no affinity to estrogen receptors α and β (ERα/β), progesterone (PR) or androgen receptor (AR) [10,16,18,19], but weak affinity towards the aryl hydrocarbon receptor (AhR) [20,21]. Most recently, treatment of human breast cancer MCF-7 cells with black cohosh extracts and with fractions containing cycloartane glycosides and cinnamic acid esters, respectively, resulted in antiproliferative effects and induction of apoptosis [22,23]. Incubation of MCF-7 cells with a fraction containing the cycloartane glycosides led to cell cycle arrest at the G1/S- and, to a lesser degree, at the G2/M-transition points, and treatment with actein affected expression levels of some proteins, such as p21, in a G1-arresting manner [24].

Finally, irrespective of the putative estrogenic activity recent investigations reporting dopaminergic [16,25], serotoninergic [26,27] and opioidic action [28] of black cohosh extracts provide evidence, that the beneficial effects such as reduction of hot flashes may be due to neurotransmitter and CNS activity.

Despite all these studies, the mode of action of black cohosh extracts and the nature of the active principles are presently not clear.

A major limitation of all these studies was that they were always conducted in a very specific perspective, while other possible modes of action were ignored. With the emergence of microarrays as a mature technology observation of global drug effects at gene expression level has become possible. This led us to perform a genome-wide gene expression profiling experiment to measure global effects of black cohosh at the mRNA level. For the study we used the ERα-positive human breast cancer cell line MCF-7, a widely used *in vitro *model for investigations of (anti)estrogenic effects and estrogen-dependent breast cancer. MCF-7 cells were treated with a lipophilic black cohosh extract. The rationale behind using this extract was that ligands for nuclear receptors such as the estrogen receptors are lipophilic molecules. Parallel experiments were carried out with 17β-estradiol (E2) and the estrogen receptor antagonist tamoxifen to compare expression profiles with these drugs. Gene expression was determined using whole-genome Affymetrix GeneChip^® ^Human Genome U133 Plus 2.0 Array, which represents about 38500 genes. Microarray results for selected genes were confirmed by real-time RT-PCR analysis. This method was also used to analyze the effects of actein, the major cycloartane glycoside in the rhizomes, and a mixture of cycloartenol aglycons. The latter was prepared to mimic a likely enzymatic hydrolysis of the glycosides in the gastrointestinal tract prior to absorption.

## Methods

### Extract

Rhizomes of black cohosh (*Cimicifugae racemosae rhizoma*) were obtained from Caesar&Loretz (Hilden, Germany, lot number 82044149). Powdered rhizome (0.5 mm particle size, 10 g) were subjected to pressurized liquid extraction using an ASE^®^200 Accelerated Solvent Extractor (Dionex, Sunnyvale, CA) at 120 bar and 70°C. After a preheating step (5 min), the sample was defatted with petroleum ether (5 min), followed by extraction with dichloromethane for 10 min. Solutions were collected in different vials. The petroleum ether solution was discarded. After evaporation of the solvent, 350 mg of dry dichloromethane extract was obtained.

### Compounds

The mixture of cycloartenol aglycons was obtained as follows: A dichloromethane extract of black cohosh rhizome (2 kg) was prepared by maceration at room temperature for 24 h. The extract (25.5 g) was separated on a silica gel column (40–63 μm, 70 × 5 cm i.d.) using a step gradient of CHCl_3_-MeOH-H_2_O (90:9.5:0.5), CHCl_3_-MeOH-H_2_O (70:30:3) and methanol. Fractions were combined on the basis of their TLC pattern. Fractions containing cycloartane glycosides were combined in equal proportions (250 mg each) and dissolved in methanol. After addition of 1 N HCl, the solution was refluxed for 1 h. Aglycons were extracted by partitioning with chloroform. The chloroform extract was evaporated to dryness, and the residue (340 mg) was purified by chromatography on a Sephadex^® ^LH20 (Pharmacia, Uppsala, Sweden) column, eluted with methanol to afford 120 mg of aglycon mixture.

Actein was purchased from Phytoplan (Heidelberg, Germany), 17β-estradiol from Sigma-Aldrich (Taufkirchen, Germany) and tamoxifen from MP Biomedicals (Eschwege, Germany).

For subsequent experiments extract and compounds were dissolved in dimethyl sulfoxide (DMSO) and diluted with appropriate assay media. The final concentration of DMSO did not exceed 0.1%. This concentration was also used for control experiments.

### Cell culture

The human breast adenocarcinoma cell line MCF-7 (obtained from ATCC) was routinely cultured in Dulbecco's Modified Eagle's Medium (DMEM) containing 10% fetal bovine serum (FBS) and Penicillin/Streptomycin (100 U/100 μg/ml) (Biochrom, Berlin, Germany) in a humidified incubator at 37°C and 5% CO_2_. Before reaching confluence, cells were splitted every 3–4 days in a 1:4- to 1:6-ratio. All experiments were performed with cells at passage numbers 25–33.

Hormone-free charcoal-dextran stripped serum (CSS) was prepared from FBS by agitating with 0.5% charcoal (Norit A) (Serva Feinbiochemica, Heidelberg, Germany) and 0.05% Dextran-T70 (Pharmacia) at 37°C for 60 min. After centrifugation at 3500 rpm CSS was filter-sterilized (0.22 μm) twice and stored at -20°C.

### Proliferation assay

MCF-7 cells were seeded in 96-well microtitre plates (Greiner Bio-One, Frickenhausen, Germany) with a density of 3500 cells per well under culture conditions and incubated at 37°C and 5% CO_2_. After 24 hours medium was removed, cells were washed with phosphate-buffered saline (PBS), and phenol red-free DMEM (Gibco™, Invitrogen, Karlsruhe, Germany) supplemented with 10% CSS and test substances in concentrations indicated in the text were added. Medium was exchanged after 72 hours. After 120 hours cells were quantified by MTT assay [29,30] as follows. Medium was removed and 100 μl MTT (Fluka, Buchs, Switzerland), dissolved in phenol red-free DMEM at 0.5 mg/ml, were added to each well. The plates were incubated at 37°C and 5% CO_2 _for 4 hours. Formazan crystals were solubilized by addition of 100 μl of 20% sodium dodecylsulfate (SDS) in H_2_O followed by incubation overnight at 37°C. Optical density was measured at 544 nm using a microplate reader (Galaxy FluoStar, BMG Labtechnologies, Offenburg, Germany) with background substraction. Relative proliferation values were calculated as percentage of negative control (0.1% DMSO value = 100%). Experiments were repeated at least three times with 4 to 6 replicates per test concentration. The results are given as mean value ± standard deviation (SD). Data were analyzed by Student's t-test. Statistically significance *vs*. DMSO control is represented as *(p < 0.05), **(p < 0.01) or ***(p < 0.001).

The acute cytotoxic potential of black cohosh extract, actein and the aglycons was determined with the Cytotoxicity Detection Kit (LDH) (Roche Diagnostics, Mannheim, Germany). No acute cytotoxicity could be found for the extract at concentrations of up to 70 μg/ml, and up to 100 μM for actein and the aglycons, respectively.

### RNA isolation

MCF-7 cells were seeded in 75 cm^2 ^culture flasks (Greiner Bio-One) at a density of 20000 cells/cm^2 ^under culture conditions and incubated at 37°C and 5% CO_2_. After 20 hours medium was removed and cells were washed with PBS. Phenol red-free DMEM containing 10% CSS and 0.1% DMSO, 15 μg/ml black cohosh extract, 20 μM actein, 30 μM aglycons, 1 nM 17β-estradiol or 10 μM tamoxifen, respectively, were added. Cells were incubated for 24 hours at 37°C and 5% CO_2_. Cells were washed twice with PBS and total RNA was extracted from cells using the RNeasy Mini Kit (Qiagen, Hilden, Germany) including on column DNase digestion according to the standard protocol. Quantity and purity of the obtained total RNA samples were determined by UV spectroscopy. RNA quality was controlled by gel electrophoresis on 1.5% agarose, stained with ethidium bromide (1 μg/ml). RNA was stored at -80°C until use.

### Array hybridization

Total RNA from each sample was labelled and hybridized to an Affymetrix GeneChip^® ^Human Genome U133 Plus 2.0 array (Affymetrix, Santa Clara, CA) according to the manufacturer's protocol (manual version 700217 rev 3), with minor modification. Briefly, 5 μg of total RNA was converted into cDNA by using the T7-(dT)_24 _Primer (Affymetrix Inc), T4gp32 (USB Corporation, Cleveland, OH) and the SuperScript II reverse transcriptase for cDNA synthesis (Invitrogen). Double-stranded cDNA was synthesized, cleaned and extracted with phenol/chloroform, followed by ethanol precipitation. Resulting cDNA was resuspended in 12 μl RNase-free water. From the ds-cDNA template biotin-labeled cRNA was made with the Enzo High Yield RNA Transcript labeling kit (Enzo Diagnostics, Farmingdale, NY). The labeled cRNA was purified following the Qiagen RNeasy Clean up protocol (Qiagen) and concentrated to 24 μl by ethanol precipitation. Fragmentation of the biotinylated cRNA (20 μg) was done directly before hybridization to the microarray. Hybridizations and scanning were performed at the Affymetrix GeneChip Core facility of the Medical Faculty, University of Leipzig. On-chip labeling was done with phycoerythrin-conjugated streptavidin. After final washing steps fluorescence was detected with the Affymetrix GeneChip 3000 7G scanner.

### Primary data analysis

Initial data analysis was performed using the Affymetrix Microarray Suite v5.1 software, setting the scaling of all probe sets to a constant value of 500 for each GeneChip. Because of the better implemented normalisation algorithm data were further normalized using RMA-Express software [31-33]. With RMAExpress all arrays were visually checked. There were no obvious failures on the chips. RMAExpress is using an enhanced quantile normalization [31,32]. All 8 experiments (4 different treatments – black cohosh extract, estradiol, tamoxifen and DMSO – in duplicate) were normalized together. Data were then exported to MS-Excel sheets. We used both the log as well as the natural output. All further comparisons and analyses are based on these RMAExpress outputs. Clearly regulated (= differentially expressed) genes were determined using the following criteria: (I) The signal intensity is higher than the median of all signal intensities on the array and (II) in both independent duplicate experiments the fold change *vs*. DMSO control is larger than 1.5.

### Real-time RT-PCR

Expression levels of 14 selected genes were determined by a two step real-time RT-PCR using the LightCycler^® ^system (Roche). Total RNA (5 μg) from each sample was transcribed into cDNA with SuperScript III reverse transcriptase (Invitrogen) and a 1:1-mixture of random hexamers (Promega, Mannheim, Germany) and oligo-dT(20) primers (MWG Biotech, Ebersberg, Germany) following the standard protocol. Resulting cDNAs were diluted to a total volume of 100 μl with water (PCR-grade). The reaction mixture for real-time PCR consisted of LightCycler^® ^Fast Start DNA Master^PLUS ^SYBR Green I Master Mix (Roche), cDNA and 0,5 μM specific forward and reverse primers (MWG Biotech) for the genes listed in Table 1. The protocol included an initial denaturation step at 95°C for 10 min, followed by 5 cycles at 95°C for 5 s, 57°C for 10 s and 72°C for 17 s and finally 45 cycles at 95°C for 5 s, 59°C for 10 s and 72°C for 17 s. Ratios between sample and control experiments were calculated after normalization of expression values to the housekeeping gene *glyceraldehyde-3-phosphate dehydrogenase *(*GAPDH*). All RT-PCR expression values were determined at least three times from 2 independent biological experiments (treatments in duplicate). Data were analyzed by Student's t-test.

**Table 1:** Primers used for real-time RT-PCR

Gene	Primer Sequence 5'-3'		Product Size
Baculoviral IAP repeat-containing 5 (BIRC5)	for	AAAGCATTCGTCCGGTTG	152 bp
	rev	CCGCAGTTTCCTCAAATTCT	
Cyclin E2 (CCNE2)	for	GACTGCTGCTGCCTTGTG	151 bp
	rev	AAAAGTCTTCAGCTTCACTGGA	
Cyclin G2 (CCNG2)	for	CCCAGAACCTCCACAACAG	158 bp
	rev	GGTGCACTCTTGATCACTGG	
Cytochrome P450, family 1, subfamily A, polypeptide 1 (CYP1A1)	for	TCTTCGCTACCTACCCAACC	196 bp
	rev	ATCTGACAGCTGGACATTGG	
Cytochrome P450, family 1, subfamily B, polypeptide 1 (CYP1B1)	for	AGAACGTACCGGCCACTATC	175 bp
	rev	GGCTGGTCACCCATACAAG	
DNA-damage-inducible transcript 4 (DDIT4)	for	GTTTGACCGCTCCACGAG	166 bp
	rev	CATCAGGTTGGCACACAAGT	
DnaJ (Hsp40) homolog, subfamily B, member 9 (DNAJB9)	for	GGATGCTGAAGCAAAATTCA	150 bp
	rev	AATGACTGCTCAAAAGAACTTCC	
E2F transcription factor 2 (E2F2)	for	CGCATCTATGACATCACCAAC	157 bp
	rev	TGTTCATCAGCTCCTTCAGC	
Estrogen receptor, alpha (ESR1)	for	CAGACACTTTGATCCACCTGA	179 bp
	rev	CTCCAGCAGCAGGTCATAGA	
GREB1 protein (GREB1)	for	ATCATCCTGAACGTGGACCT	151 bp
	rev	CCACGATCTGCTTCTTCATC	
Growth arrest and DNA-damage-inducible, alpha (GADD45A)	for	GGAGGAAGTGCTCAGCAAA	169 bp
	rev	CTGGATCAGGGTGAAGTGG	
Metastasis associated in lung adenocarcinoma transcript 1 (MALAT-1)	for	TGCAATTTGGTGATGAAGGT	161 bp
	rev	CAACATATTGCCGACCTCAC	
Proliferating cell nuclear antigen (PCNA)	for	TTGCACTGAGGTACCTGAACTT	160 bp
	rev	CCTTCTTCATCCTCGATCTTG	
Vascular endothelial growth factor (VEGF)	for	CATCTTCAAGCCATCCTGTG	179 bp
	rev	TGCATTCACATTTGTTGTGC	
Glyceraldehyde-3-phosphate dehydrogenase (GAPDH)	for	ACCAGGTGGTCTCCTCTGAC	173 bp
	rev	TTACTCCTTGGAGGCCATGT	

## Results

### Proliferation assay

**Figure 2:**
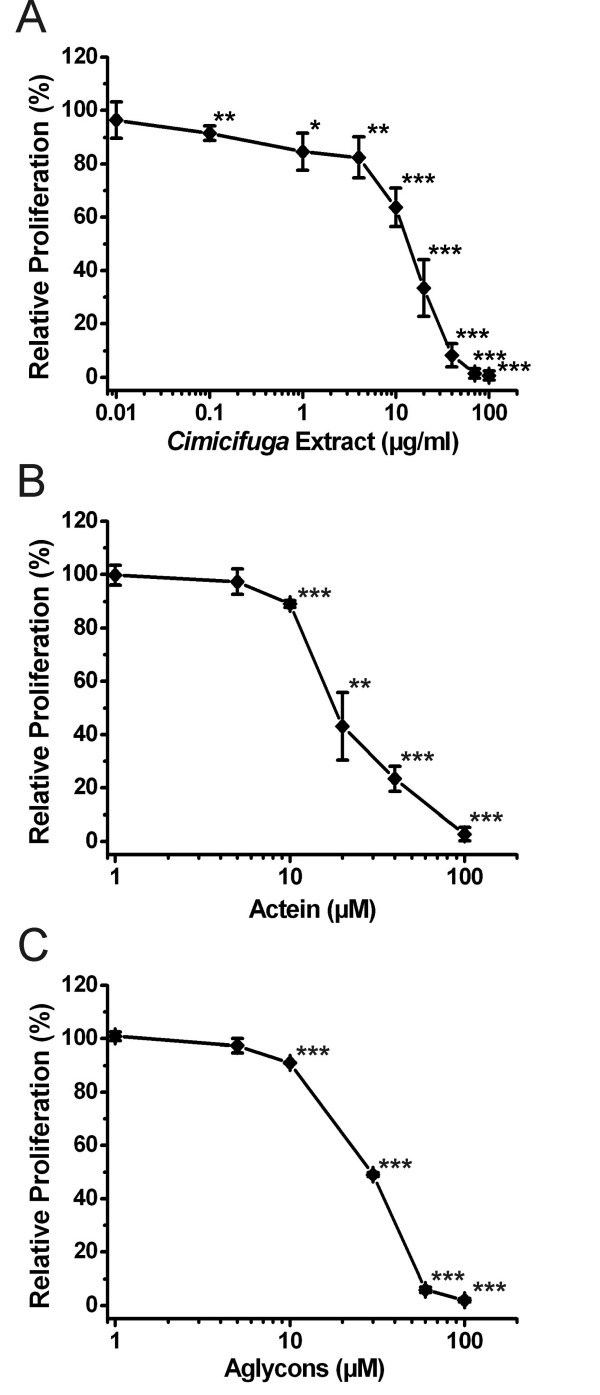
Proliferation assay: Effects on growth rate of MCF-7 cells. MCF-7 cells were treated with (**A**) dichloromethane extract of black cohosh rhizome, (**B**) actein or (**C**) mixture of aglycons derived from cycloartane glycosides. Controls (100% proliferation) contained 0.1% DMSO. After 120 h cell number was determined by MTT dye-reduction assay. Relative proliferation data are presented as means ± SD (n = 3–6). *p < 0.05, **p < 0.01, ***p < 0.001 *vs*. control (Student's t-test).

We first performed a MTT-based proliferation assay to explore how the black cohosh extract and the selected compounds influence growth of MCF-7 cells. 17β-estradiol, the positive control, significantly stimulated cell proliferation after 120 h, reaching a plateau of maximal stimulation (184.2 ± 28.1% *vs*. DMSO control; p < 0.001) at a concentration of 1 nM. The magnitude was comparable to effects observed in other studies with MCF-7 (ATCC) cells [11,34,35]. At a concentration of 10 μM tamoxifen reduced cell proliferation to 45.7 ± 6.8% (p < 0.001 *vs*. DMSO control). Exposure of MCF-7 cells for 120 h to different concentrations of black cohosh extract, the cycloartane glycoside actein and the cycloartane aglycon mixture all inhibited cell proliferation in a dose-dependent manner (Figure 2). IC_50 _values – the concentrations that caused 50% inhibition of growth – were determined by extrapolation for black cohosh extract (14.7 ± 2.6 μg/ml), actein (19.6 ± 3.1 μM) and the aglycon mixture (30.3 ± 0.5 μM). These proliferation and cytotoxicity measurements provide only a preliminary information of biological activity.

### Gene expression microarray analysis with black cohosh extract

#### General results

For gene expression profiling MCF-7 cells were treated with 15 μg/ml black cohosh extract, 1 nM 17β-estradiol, 10 μM tamoxifen or DMSO control for 24 h in the presence of 10% charcoal stripped serum. After RNA extraction, gene expression profiles were recorded using Affymetrix HG U133 Plus 2.0 GeneChip Arrays as described. Regulated (differentially expressed) genes were identified using the following selection criteria: minimal signal intensity > median and fold change *vs*. control > 1.5 in two independent experiments. With these criteria we identified 544 probe sets (~1% from the more than 54000 probe sets on the HG U133 Plus 2.0 Array) representing 431 genes regulated by the extract. The false positive rate with the criteria used is about 5% based on random permutation analysis of the gene expression results, showing a mean of 22 to 32 regulated probe sets (22/544 ~ 4.0%; 32/544 ~ 5.9%).

**Figure 3:**
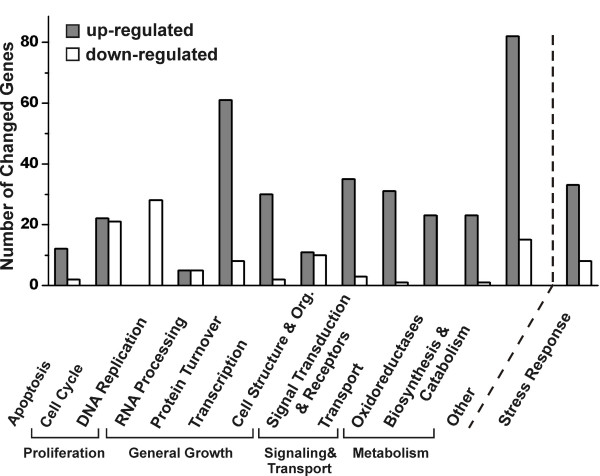
Functional categories of genes regulated in MCF-7 cells after 24 h incubation with black cohosh extract. Genes were grouped in 5 large groups (*Apoptosis*, *Proliferation*, *General Growth*, *Signaling & Transport*, *Metabolism*), some consisting of subgroups. Genes that are not clearly associated with these groups are summarized in the category *others*. The category *stress response *contains genes also grouped into one of the 6 main classes. Each bar represents the number of genes that were up- (dark) or downregulated (white) in the respective group.

Of the genes regulated by the extract, 335 transcripts (78%) were upregulated and 96 genes (22%) were downregulated. These genes were grouped into functional categories according to Gene Ontology terms and gene description at the NetAffx™ Analysis Center [36] and addition literature search. Most of the regulated genes could be clearly assigned to 5 larger groups of functionally related genes (subgroups in brackets): *apoptosis*, *proliferation *(*cell cycle*, *replication*), *general growth *(*RNA processing*, *protein turnover*, *transcription*, *cell structure and organization*), *signaling & transport *(*signal transduction*, *transport*) and *metabolism *(*oxidoreductases*, *biosynthesis and catabolism*). Genes that could not be assigned to any of these groups were summarized as *others *(Figure 3). Furthermore, because of a striking presence of many transcripts linked to cellular stress response, we also created the functional category *stress response*, which is not directly linked to the other categories. Genes functionally connected to this group are already members of one of the 6 main categories (Figure 3). In most groups more genes were stimulated than inhibited. In the group *proliferation*, in contrast, a majority of genes appeared to be downregulated. Genes for this category as well as the groups/subgroups *apoptosis*, *protein turnover, oxidoreductases and stress response *are all statistically overrepresented among the black cohosh regulated genes (p < 0.001, two-sided p-value Fisher Exact Test). For two groups – *proliferation *(*cell cycle*, *DNA replication*) and *stress response *– the overrepresentation is very highly significant.

Regulation of 13 genes representing the 6 functional categories, *apoptosis*, *proliferation*, *general growth*, *signaling & transport*, *metabolism *and *others*, were verified by real-time RT-PCR from independent samples to confirm the regulation of genes connected to the major cellular effects observed.

**Figure 4:**
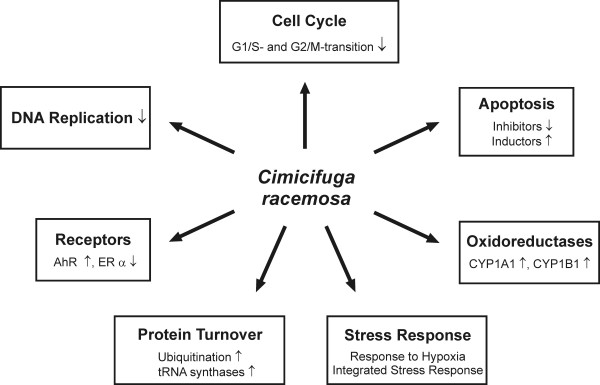
Summary of effects of black cohosh in MCF-7 cells at mRNA level observed with the microarray experiment. ↓ represents inhibition, ↑ represents stimulation.

The cellular effects of the functional groups regulated by black cohosh are summarized in Figure 4. Expression values of selected genes are presented in Table 2. A complete list of all regulated genes is available [see Additional file 1]. The data discussed in this publication have also been deposited in NCBIs Gene Expression Omnibus (GEO) [37], and are accessible through GEO Series accession number GSE6800.

**Table 2:** List of selected genes regulated by black cohosh extract in MCF-7 cells. Genes are listed with symbol, GenBank accession number and fold changes *vs*. DMSO control of two independent microarray experiments. Genes in bold are associated to stress response. Those written in *italics *have been verified by real-time RT-PCR. A full list with all 431 genes is available [see Additional file 1].

**Gene Title**	**Gene Symbol**	**Accession No.**	**Fold Change**	
			**#1**	**#2**
**APOPTOSIS**				
***DNA-damage-inducible transcript 4***	***DDIT4***	NM_019058	5.1	5.6
**P8 protein (candidate of metastasis 1)**	**P8**	AF135266	4.4	2.7
Tumor protein p53 inducible nuclear protein 1	TP53INP1	AW341649	3.5	3.1
Tumor necrosis factor receptor superfamily, member 10b	TNFRSF10B	AF016266	2.5	2.7
**Immediate early response 3**	**IER3**	NM_003897	2.0	1.9
**BCL2/adenovirus E1B 19 kda interacting protein 3-like**	**BNIP3L**	AL132665	1.8	1.6
Helicase, lymphoid-specific	HELLS	AI650364	-2.8	-2.7
*Baculoviral IAP repeat-containing 5 (survivin)*	*BIRC5*	AA648913	-3.1	-2.0
				
**PROLIFERATION**				
**Cell cycle/Proliferation**				
Growth differentiation factor 15	GDF15	AF003934	6.0	4.5
***Vascular endothelial growth factor***	***VEGF***	AF022375	4.6	4.2
*Cyclin G2*	*CCNG2*	AW134535	2.8	2.3
**DNA-damage-inducible transcript 3 (CHOP-10, GADD153)**	**DDIT3**	BC003637	2.6	2.7
RAS, dexamethasone-induced 1	RASD1	AF069506	2.6	2.4
**Sestrin 2**	**SESN2**	BF131886	2.4	2.8
***Growth arrest and DNA-damage-inducible, alpha***	***GADD45A***	NM_001924	2.2	2.6
**Cyclin-dependent kinase inhibitor 1A (p21, Cip1)**	**CDKN1A**	NM_000389	1.8	1.6
Forkhead box O3A	FOXO3A	AV725666	1.8	1.9
Cyclin B1 interacting protein 1	CCNB1IP1	NM_021178	1.6	1.6
Cyclin-dependent kinase 7	CDK7	L20320	1.6	1.6
Cyclin F	CCNF	U17105	-1.5	-1.9
S-phase kinase-associated protein 2 (p45)	SKP2	BC001441	-1.6	-1.6
**Chromatin assembly factor 1, subunit A (p150)**	**CHAF1A**	BF062223	-1.7	-1.6
Membrane-associated tyrosine- and threonine-specific cdc2-inhibitory kinase	PKMYT1	NM_004203	-1.8	-1.6
*E2F transcription factor 2*	*E2F2*	AL561296	-2.2	-1.6
Kinetochore protein Spc24	Spc24	AI469788	-2.2	-1.8
Cyclin A2	CCNA2	NM_001237	-2.3	-1.5
Proliferation-related Ki-67 antigen	MKI67	AU152107	-2.3	-1.9
*Cyclin E2*	*CCNE2*	AF112857	-2.6	-3.0
E2F transcription factor 7	E2F7	AI341146	-2.7	-2.1
Kinesin family member 11 (Eg5)	KIF11	NM_004523	-2.7	-1.7
**DNA replication, repair and synthesis**				
**Recq protein-like 4**	**RECQL4**	NM_004260	-1.7	-1.7
Cyclin-dependent kinase 2	CDK2	AB012305	-2.1	-1.5
Minichromosome maintenance deficient 3 (S. Cerevisiae)	MCM3	NM_002388	-2.1	-1.8
CDC6 cell division cycle 6 homolog (S. Cerevisiae)	CDC6	NM_001254	-2.5	-1.6
DNA replication factor	CDT1	AF321125	-2.5	-1.7
**BRCA1 interacting protein C-terminal helicase 1**	**BRIP1**	AF360549	-2.6	-1.7
Minichromosome maintenance deficient 5, cell division cycle 46 (S. Cerevisiae)	MCM5	AA807529	-2.6	-1.7
Minichromosome maintenance deficient 7 (S. Cerevisiae)	MCM7	AF279900	-2.7	-1.8
Ubiquitin-like, containing PHD and RING finger domains, 1	UHRF1	AK025578	-2.7	-2.7
Thymidine kinase 1, soluble	TK1	BC007986	-2.8	-1.7
***Proliferating cell nuclear antigen***	***PCNA***	NM_002592	-2.9	-1.9
**Replication factor C (activator 1) 3, 38 kda**	**RFC3**	NM_002915	-3.0	-1.7
Minichromosome maintenance deficient 2, mitotin (S. Cerevisiae)	MCM2	NM_004526	-3.1	-1.6
DNA replication complex GINS protein PSF2	Pfs2	BC003186	-3.3	-1.5
**Flap structure-specific endonuclease 1**	**FEN1**	NM_004111	-3.4	-2.5
Minichromosome maintenance deficient 4 (S. Cerevisiae)	MCM4	AI859865	-3.4	-2.4
Thymidylate synthetase	TYMS	AB077208	-3.4	-2.4
ASF1 anti-silencing function 1 homolog B (S. Cerevisiae)	ASF1B	NM_018154	-3.5	-2.1
				
**GENERAL GROWTH**				
**Protein processing**				
**Chromosome 1 open reading frame 24**	**C1orf24**	AF288391	4.9	5.1
Seryl-tRNA synthetase	SARS	AU147785	4.4	4.0
DnaJ (Hsp40) homolog, subfamily C, member 10	DNAJC10	BG168666	3.1	1.9
*DnaJ (Hsp40) homolog, subfamily B, member 9*	*DNAJB9*	AL080081	2.9	2.3
**Homocysteine-inducible, endoplasmic reticulum stress-inducible, ubiquitin-like domain member 1**	**HERPUD1**	AF217990	2.5	2.0
Tryptophanyl-tRNA synthetase	WARS	NM_004184	2.5	2.2
**MAP kinase-interacting serine/threonine kinase 2**	**MKNK2**	NM_017572	2.4	2.0
Eukaryotic translation initiation factor 4E binding protein 1	EIF4EBP1	AB044548	2.3	1.8
**Stress 70 protein chaperone, microsome-associated, 60 kda**	**STCH**	NM_006948	2.3	2.7
**Eukaryotic translation initiation factor 2-alpha kinase 3 (syn. PERK)**	**EIF2AK3**	NM_004836	2.2	1.7
Methionine-tRNA synthetase	MARS	AA621558	2.2	2.2
Microtubule-associated protein 1 light chain 3 beta	MAP1LC3B	AF183417	2.2	1.9
Tyrosyl-tRNA synthetase	YARS	AW245400	2.0	1.7
Cysteinyl-tRNA synthetase	CARS	AI769685	1.9	2.0
Isoleucine-tRNA synthetase	IARS	NM_013417	1.9	2.0
F-box only protein 11	FBXO11	AL117620	1.9	1.7
Ubiquitin protein ligase E3 component n-recognin 1	UBR1	AV715153	1.9	1.8
Glutamyl-prolyl-tRNA synthetase	EPRS	NM_004446	1.8	1.7
**Heat shock 70 kda protein 5 (glucose-regulated protein, 78 kda)**	**HSPA5**	AF216292	1.8	1.6
Transducin (beta)-like 1X-linked	TBL1X	AW968555	1.8	1.9
Ubiquitin specific protease 3	USP3	AF077040	1.8	1.8
**Eucaryotic translation initiation factor 1**	**EIF1**	AF083441	1.7	1.7
Glycyl-tRNA synthetase	GARS	D30658	1.7	2.3
SUMO1/sentrin specific protease 6	SENP6	AF306508	1.7	1.6
**APG12 autophagy 12-like (S. Cerevisiae)**	**APG12L**	BE965998	1.6	1.7
Ubiquitin-fold modifier 1	Ufm1	NM_016617	1.6	1.6
**Heat shock 70 kda protein 2**	**HSPA2**	U56725	-1.7	-1.9
F-box only protein 5 (early mitotic inhibitor 1)	FBXO5	AK026197	-2.0	-1.7
Ubiquitin-conjugating enzyme E2C	UBE2C	NM_007019	-2.0	-1.6
**Transcription**				
**Basic helix-loop-helix domain containing, class B, 2**	**BHLHB2**	NM_003670	3.6	2.6
Trinucleotide repeat containing 9	TNRC9	AK025084	3.4	2.1
Cbp/p300-interacting transactivator, with Glu/Asp-rich carboxy-terminal domain, 2	CITED2	AF109161	3.1	1.5
**CCAAT/enhancer binding protein (C/EBP), beta**	**CEBPB**	AL564683	2.4	2.3
CCAAT/enhancer binding protein (C/EBP), gamma	CEBPG	NM_001806	2.2	2.4
Junction-mediating and regulatory protein	JMY	BF447037	2.2	1.6
**Endothelial PAS domain protein 1 (Hypoxia-inducible factor 2, alpha)**	**EPAS1**	AF052094	2.1	1.9
**Nuclear factor (erythroid-derived 2)-like 2**	**NFE2L2**	NM_006164	2.0	1.5
**Activating transcription factor 3**	**ATF3**	NM_001674	1.8	2.0
**Activating transcription factor 4**	**ATF4**	NM_001675	1.7	1.5
**Early growth response 1**	**EGR1**	AI459194	1.7	4.7
**Nuclear factor (erythroid-derived 2)-like 1**	**NFE2L1**	NM_003204	1.6	1.6
Chromobox homolog 4 (Pc class homolog, Drosophila)	CBX4	AI570531	1.5	1.8
Kruppel-like factor 4 (gut)	KLF4	BF514079	1.5	1.8
Zinc finger protein 36, C3H type-like 2	ZFP36L2	AI356398	-3.1	-2.1
**Cell organization, adhesion & structure and cytoskeleton**				
**Decay accelerating factor for complement (CD55, Cromer blood group system)**	**DAF**	NM_000574	3.6	3.3
Ras homolog gene family, member B	RHOB	AI263909	-1.8	-1.6
				
**SIGNALING AND TRANSPORT**				
**Receptors & Signal transduction**				
Unc-5 homolog B (C. Elegans)	UNC5B	AK022859	3.3	2.1
Stanniocalcin 2	STC2	BC000658	2.6	2.3
TRAF family member-associated NFKB activator	TANK	U59863	2.2	2.3
**Interferon gamma receptor 1**	**IFNGR1**	NM_000416	2.1	1.6
**Mitogen-inducible gene 6**	**MIG-6**	AL034417	2.1	2.5
Ras association (ralgds/AF-6) domain family 3	RASSF3	AI628605	2.0	1.5
**Aryl hydrocarbon receptor**	**AHR**	NM_001621	1.8	1.8
Tribbles homolog 1 (Drosophila	TRIB1	NM_025195	1.7	1.6
Tribbles homolog 3 (Drosophila)	TRIB3	NM_021158	1.7	1.6
Aryl hydrocarbon receptor nuclear translocator-like	ARNTL	AB000815	1.6	1.8
Casein kinase 1, gamma 3	CSNK1G3	NM_004384	1.5	1.6
**Hypoxia-inducible factor 1, alpha subunit**	**HIF1A**	NM_001530	1.5	1.5
**c-Jun N-terminal kinase 1 (mitogen-activated protein kinase 8, MAPK8)**	**JNK1**	AU152505	1.5	1.5
Tyrosine 3-monooxygenase/tryptophan 5-monooxygenase activation protein, eta polypeptide	YWHAH	NM_003405	-1.8	-1.9
*Estrogen receptor, alpha*	*ESR1*	NM_000125	-2.0	-1.8
**Transport**				
Solute carrier family 7, (cationic amino acid transporter, y+ system) member 11	SLC7A11	AA488687	6.2	5.7
Potassium voltage-gated channel, Isk-related family, member 4	KCNE4	AI002715	3.1	2.1
Solute carrier family 7 (cationic amino acid transporter, y+ system), member 5	SLC7A5	AB018009	2.3	2.0
**Solute carrier family 38, member 2**	**SLC38A2**	NM_018976	2.0	2.0
Ferritin H	FTH1	AA083483	1.9	1.9
**Solute carrier family 1 (glutamate/neutral amino acid transporter), member 4**	**SLC1A4**	AI889380	1.6	1.6
				
**METABOLISM**				
**Oxidoreductase activity**				
*Cytochrome P450, family 1, subfamily A, polypeptide 1*	*CYP1A1*	NM_000499	12.4	3.5
Aldo-keto reductase family 1, member C1 (20-alpha (3-alpha)-hydroxysteroid dehydrogenase)	AKR1C1	M33376	5.5	2.3
Aldehyde dehydrogenase 1 family, member L2	ALDH1L2	AI654224	5.0	2.2
*Cytochrome P450, family 1, subfamily B, polypeptide 1*	*CYP1B1*	NM_000104	4.1	2.7
**Heme oxygenase (decycling) 1**	**HMOX1**	NM_002133	3.3	2.1
Glutaredoxin (thioltransferase)	GLRX	AF162769	3.0	1.9
Sterol-C4-methyl oxidase-like	SC4MOL	AV704962	2.5	2.4
Aspartate beta-hydroxylase	ASPH	AF289489	2.4	2.2
5-Methyltetrahydrofolate-homocysteine methyltransferase reductase	MTRR	NM_024010	2.3	2.2
Methylene tetrahydrofolate dehydrogenase (NAD+ dependent)	MTHFD2	NM_006636	1.9	2.0
Sterol-C5-desaturase	SC5DL	D85181	1.5	1.6
Cytochrome P450, family 51, subfamily A, polypeptide 1 (Sterol 14-alpha-demethylase)	CYP51A1	NM_000786	1.6	1.6
Farnesyl-diphosphate farnesyltransferase 1 (Squalene synthase)	FDFT1	AA872727	1.6	1.7
3-Hydroxy-3-methylglutaryl-Coenzyme A reductase	HMGCR	AL518627	1.8	1.7
**Biosynthesis/Catabolism**				
**Asparagine synthetase**	**ASNS**	NM_001673	3.7	4.1
Acyl-coa synthetase long-chain family member 1	ACSL1	NM_021122	2.6	2.2
Phosphoenolpyruvate carboxykinase 2 (mitochondrial)	PCK2	NM_004563	2.5	3.3
Spermidine/spermine N1-acetyltransferase	SAT	BE326919	1.6	1.8
				
**OTHERS**				
*Metastasis associated in lung adenocarcinoma transcript 1*	*MALAT-1*	BG534952	11.0	3.1
S100 calcium binding protein P	S100P	NM_005980	5.8	5.1
Gastric-associated differentially-expressed protein YA61P, drug sensitive protein 1	---	AF220415	5.6	2.1
Hypothetical protein LOC222171	LOC222171	AI347918	5.1	3.6
Brain expressed X-linked 2	BEX2	AF251053	4.6	3.6
Myozenin 2 (calcisarcin 1)	MYOZ2	AI475544	4.5	3.8
WD40 repeat protein Interacting with phosphoinositides of 49 kda	WIPI49	AW052084	3.5	2.1
SLIT and NTRK-like family, member 6	SLITRK6	AI680986	3.4	2.4

#### Proliferation

**Figure 5:**
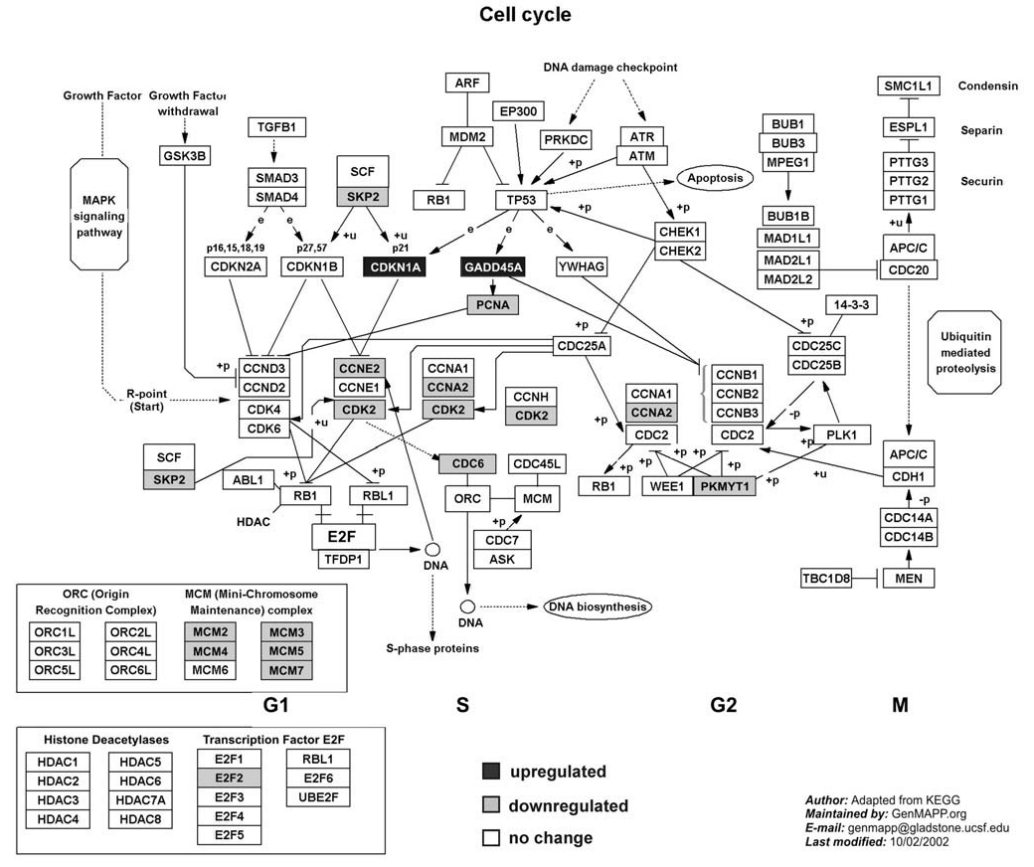
Cell cycle pathway diagram obtained from GenMAPP (Gene Map Annotator and Pathway Profiler, Gladstone Institutes, University of San Francisco, San Francisco, CA [38]. Proteins involved in cell cycle control are displayed from left to right as cell cycle progresses from G1 through S and G2 to M-phase. Genes with boxes marked black were upregulated. Genes marked with grey boxes were downregulated.

In agreement with the anti-proliferative effect of the black cohosh extract, genes involved in proliferation control were significantly overrepresented. Transcripts related to cell cycle regulation and DNA replication were regulated in a manner supporting cell cycle arrest. Genes, whose products are involved in the transition from G1 to S-phase appeared to be downregulated, such as cyclins (*CCNA2*, *CCNE2*, *CCNF*), *cyclin-dependent kinase *2 (*CDK2*) and transcription regulators (*E2F2*, *PCNA*, *SKP2*), whereas transcription of inhibitory genes *cyclin G2 *(*CCNG2*), *GADD45A *(*growth arrest and DNA-damage-inducible, alpha*) and *p21*^*cip*1 ^(*cyclin-dependent kinase inhibitor 1A*, *CDKN1A*) was increased. Elevated levels of *CCNG2*, *cyclin B1 interacting protein 1 *(*CCNB1IP1*), *forkhead box O3A *(*FOXO3A*), *GADD45A *and *p21*^*cip*1 ^genes as well as downregulation of *cyclin A2 *(*CCNA2*) and *CDK2 *provided evidence that cell cycle progression might be additionally arrested at the G2/M-checkpoint. The level of various DNA replication related genes (*CDC6*, *CDT1*, *FEN1*, *MCM2*, *MCM3*, *MCM4*, *MCM5*, *MCM7*, *MCM10*, *Pfs2*, *RFC3*) was also reduced, thereby suggesting a reduction in the replication rate. All cell cycle related effects are summarized in Figure 5, which shows a cell cycle diagram from GenMAPP (Gene Map Annotator and Pathway Profiler, Gladstone Institutes, University of San Francisco, San Francisco, CA) [38], in which up- and downregulated genes are marked.

#### Apoptosis

In addition to the regulation of genes involved in proliferation control, we also observed regulation of apoptosis-linked genes in a pro-apoptotic manner, indicating that the extract could sensitize breast cancer cells for apoptotic events. An increase in apoptotic events would also contribute to the decrease in cellular proliferation observed. In cells treated with black cohosh the transcript of the apoptosis inhibitor survivin (*baculoviral IAP repeat-containing 5*, *BIRC5*) was downregulated, whereas genes coding for apoptosis-inducing and -supporting products (*BNIP3L*, *TNFRSF10B*, *TP53INP1*) were increased. *FOXO3A*, *GADD45A*, *GDF 15 *(*growth differentiation factor 15*) and *p21*^*cip*1^, whose mRNA levels increased, are also connected with apoptosis in addition to their role in cell cycle control. Transcript of tyrosyl-tRNA synthetase (YARS), whose secretion is linked to apoptotic events [39], was upregulated. Downregulation of *lymphoid-specific helicase *(*HELLS*), as observed in our experiments, has been reported to be associated with apoptosis [40]. The upregulation of *c-Jun N-terminal kinase 1 *(*JNK1 *= *MAPK8*) and *DNA-damage-inducible transcript 3 *(*DDIT3 *= *CHOP *= *GADD153*), as observed in our experiments, have been reported as related to stress-induced apoptosis [41,42]. *p8 Protein *(*p8*), *immediate early response 3 *(*IER3*) and *DNA-damage inducible transcript 4 *(*DDIT4*, also known as *REDD1 *or *RTP801*), whose transcript was strongly upregulated, are known to be expressed under cellular stress (described later) and have been associated with both pro- and anti-apoptotic events [43-46].

#### Stress response

**Figure 6:**
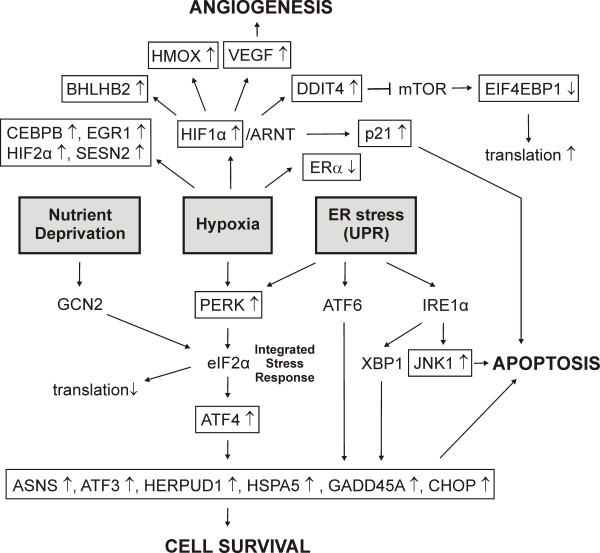
Stress response pathways affected by black cohosh at the transcriptional level. Genes marked by boxes were regulated in MCF-7 cells after treatment with black cohosh extract. ↑ represents up-regulation, ↓ represents down-regulation. (Abbreviations: ATF6, activating transcription factor 6; XBP1, x box binding protein 1; IRE1α, Serine/threonine-protein kinase/endoribonuclease, Inositol-requiring 1).

In relation to the results above, overexpression of transcripts involved with response to cellular stress was highly statistically significant after treatment with black cohosh extract. Among different functional categories we identified some 40 transcripts associated with metabolic stress response such as hypoxia [47], mal- or unfolded protein response in the endoplasmic reticulum [48,49] or starvation for amino acids or glucose [50,51] (Figure 6). Transcript of hypoxia inducible factor 1α (HIF1α; HIF1A), a key regulator in hypoxia, was upregulated. A heterodimer of HIF1α/ARNT (HIF1) binds to hypoxia-responsive elements (HREs), thereby regulating the expression of hypoxia-response genes. *Vascular endothelial growth factor *(*VEGF*), *heme oxygenase 1 *(*HMOX1*), *basic helix-loop-helix domain containing, class B, 2 *(*BHLHB2*), *p21*^*cip*1 ^and *DDIT4 *– these transcripts were also upregulated – are known to be direct target genes [47,52,53]. A hypoxia response pathway via mTOR (mammalian target of rapamycin) including inactivation of EIF4EBP1 (Eukaryotic translation initiation factor 4E binding protein 1) and finally resulting in increased mRNA translation is known to be inhibited by DDIT4 [52]. This could explain the increase of EIF4EBP1 mRNA we observed in our experiment. The increases of *CCAAT/enhancer binding protein, beta *(*CEBPB*), *endothelial PAS domain protein 1 *(*EPAS1*=*HIF2α*), *early growth response 1 *(*EGR1*) and *sestrin 2 *(*SESN2*) mRNA are also related to hypoxia [53,54].

Furthermore, we observed regulation of genes related to endoplasmic reticulum stress response (unfolded protein response, UPR), which involves the activation of three different pathways. Transcription of c-Jun N-terminal kinase 1 (JNK1 = MAPK8) was upregulated in our experiment. JNK1 is a target of one UPR-pathway and its activation may lead to apoptosis [42,55]. Phosphorylation of eukaryotic translation initiation factor 2 α (eIF2α) at Ser51 is involved not only in UPR but also in responses to hypoxia, nutrient deprivation and other cellular stresses. Hence, this evolutionarily conserved pathway has been termed the integrated stress response (ISR) [41,52]. PERK (EIF2AK3, eukaryotic translation initiation factor 2-alpha kinase *3*), whose mRNA-level was increased by black cohosh treatment, is a kinase linking hypoxia stress reponse and UPR to eIF2α-phosphorylation, whereas amino acid and glucose starvation response acts via GCN2 kinase. As a consequence of eIF2α-phosphorylation the translation of most mRNAs is inhibited, but paradoxically the translation of *activating transcription factor 4 *(*ATF4*) is increased. We observed an upregulation of the ATF4 gene as well as various ATF4-induced downstream target genes, e.g. *ASNS *(*asparagine synthetase*), *ATF3 *(*activating transcription factor 3*), *CHOP*, *GADD45A*, *HERPUD1 *(*homocysteine-inducible, endoplasmic reticulum stress-inducible, ubiquitin-like domain member 1*), *HSPA5 *(*heat shock 70 kDa protein 5*). Gene products of these transcripts are involved in cell survival and tumorigenesis as well as apoptotic events [42,49,56].

#### Protein turnover

In the context of mis- or unfolded protein response some other processes of protein turnover are affected by black cohosh extract. The expression levels of various ubiquitin cycle-related genes (*FBOX5*, *FBOX11*, *MAP1LC3B*, *SENP6*, *TBL1X*, *UBE2C*, *UBR1*, *Ufm1*, *USP3*) were influenced by black cohosh. Some of these transcripts code for products involved with cell cycle progression and were regulated in a cell cycle arresting manner, emphasizing our results described above. Inhibitory *CCNB1IP1 *was upregulated, whereas *SKP2 *(*S-phase kinase-associated protein 2*; *p45*) and UHRF1 (*Ubiquitin-like, containing PHD and RING finger domains, 1*) were downregulated. Furthermore, mRNA-levels of eight different aminoacyl-tRNA synthetases (*CARS*, *EPRS*, *GARS*, *IARS*, *MARS*, *SARS*, *WARS*, *YARS*) were upregulated.

#### Oxidoreductases

In response to black cohosh treatment, transcripts of several oxidoreductases involved with metabolism of xenobiotics were affected. In some cases we observed strong induction. The most prominent upregulation of all *Cimcifuga *regulated genes occurred for *CYP1A1 *(*cytochrome P450, family 1, subfamily A, polypeptide 1*). In the microarray experiments the increase was 12.4 and 3.5 fold, while RT-PCR gave even higher changes (28.5 ± 14.0 fold, p < 0.01 and 19.2 ± 10.9 fold, p < 0.05). This was accompanied by a marked but lower increase of *CYP1B1 *(*cytochrome P450, family 1, subfamily B, polypeptide 1*). Both enzymes are known to be activated by xenobiotics through the aryl hydrocarbon receptor pathway. Transcript of heme oxygenase 1 (HMOX1), an essential enzyme in heme catabolism and a direct target of HIF1 (see stress response), was also upregulated. Finally, transcripts of several enzymes of the cholesterol biosynthesis pathway (*CYP51A1*, *FDFT1*, *HMGCR*, *SC4MOL*, *SC5DL*) were marginally increased.

#### Receptors

Interestingly, transcription of the aryl hydrocarbon receptor (AhR) which regulates expression of the CYPs, was also increased. Furthermore, we observed a downregulation of the estrogen receptor α gene (*ESR1*). This regulation has already been reported in response to hypoxic stress [57]. mRNAs coding for interferon gamma receptor 1 (IFNGR1) appeared to be upregulated. The latter promotes effects of interferon γ, whose antitumor activity has previously been reported [58].

#### Others

Within this category, two tumor-associated transcripts showed largest upregulation. *MALAT-1 *(*metastasis in lung adenocarcinoma transcript 1*), a recently identified noncoding RNA, has been shown to be activated in early-stage lung cancer. High-level expression is associated to low survival rates of patients due to high risk to develop metastatis [59]. *S100P *(*S100 calcium binding protein P*) was reported as marker of breast cancer initiation. Expression of S100P as well as FTH1 (Ferritin H), an iron-binding protein, whose transcript was also upregulated by black cohosh, is associated to immortalization and transformation of breast epithelial cells [60].

### Comparison of the expression pattern of black cohosh with tamoxifen and E2

**Figure 7:**
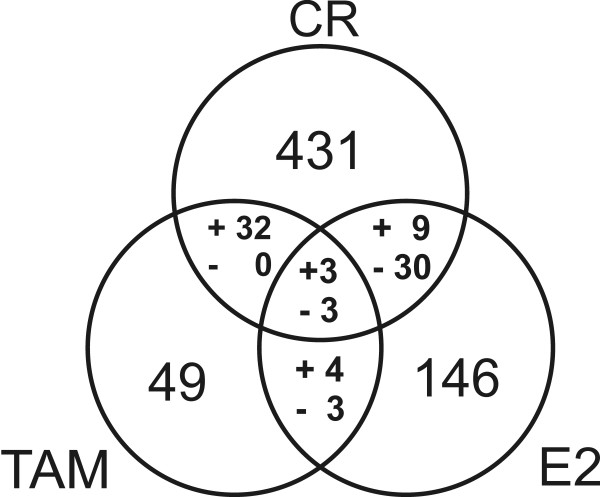
Overlap of expression profiles. Numbers within the 3 circles represent the genes that were differentially expressed according to our filters after 24 h treatment of MCF-7 cells with *Cimicifuga racemosa *(black cohosh) extract (**CR**), 17β-estradiol (**E2**) or tamoxifen (**TAM**). Numbers within the intersections represent genes regulated with both of the respective treatments or – in the middle – all 3 different treatments. For every intersection genes regulated in the same direction up or down (correlated) are marked with (**+**). Genes regulated in opposite directions (anti-correlated) are marked with (**-**).

**Figure 8:**
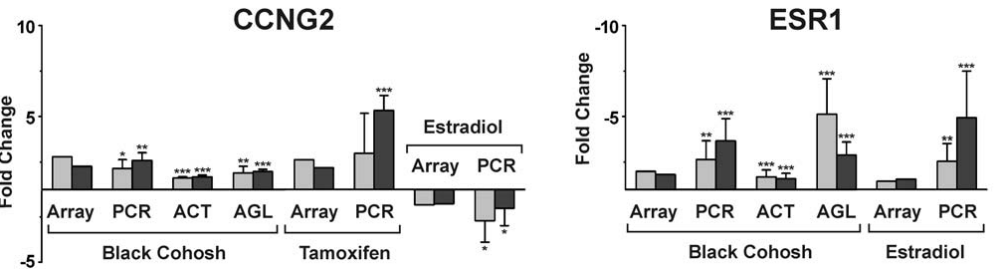
Gene expression levels of *cyclin G2 *(*CCNG2*) and *estradiol receptor α *(*ESR1*) in MCF-7 cells after 24 h treatment with black cohosh extract (15 μg/ml), the triterpene glycoside actein (20 μM), the triterpene aglycon mixture (30 μM), tamoxifen (10 μM) and estradiol (1 nM). For the treatments with extract, tamoxifen and estradiol, results obtained with microarrays (**Array**) and real-time RT-PCR (**PCR**) are shown. For actein (**ACT**) and the aglycons (**AGL**) expression levels were determined by real-time RT-PCR. Bars represent gene expression levels as fold changes calculated *versus *DMSO control. RT-PCR measurements were done at least in triplicate. The data are presented as means ± SD (*p < 0.05, **p < 0.01, ***p < 0.001: gene expression statistically significantly different from DMSO control, calculated by Student's t-test).

Expression profiles with 17β-estradiol and the estrogen receptor-antagonist tamoxifen were investigated in parallel to compare the patterns with the black cohosh extract. After treatment with 1 nM E2 146 transcripts met our selection criteria, among these known estrogen-regulated genes such as *insulin-like growth factor binding protein 4 *(*IGFBP4*) or *GREB1 protein *(*GREB1*). The latter transcript was strongly upregulated (microarray: 21.7 and 10.4 fold; RT-PCR: 26.6 ± 10.1, p < 0.05 and 15.4 ± 5.7, p < 0.05). With 10 μM tamoxifen 49 genes were observed to be regulated. Figure 7 shows the intersection of expression patterns of black cohosh, E2 and tamoxifen. Among a total of 39 genes that were regulated both under black cohosh and E2 treatment, 30 transcripts were affected in opposite directions (anti-correlated). In contrast, a comparison of black cohosh and tamoxifen revealed a correlated regulation of 32 transcripts. This is quite surprising given that only 49 genes were considered regulated in response to tamoxifen. Hence, the expression profile of black cohosh was more related to tamoxifen. Since E2 stimulated and tamoxifen inhibited proliferation of MCF-7 cells in our assay, it was not surprising that most genes in the intersections are related to cell cycle regulation and apoptosis. Among the genes associated with cell cycle arrest and apoptosis that were regulated in all treatments, the two cell cycle inhibitory transcripts *cyclin G2 *(Figure 8) and *tumor protein p53 inducible nuclear protein 1 *(*TP53INP1*) were both upregulated by black cohosh and tamoxifen and downregulated by E2. Apart from *ESR1*, which was downregulated with E2 and black cohosh treatment (Figure 8), and *VEGF*, which can be considered to be regulated via hypoxia response pathways, no gene affected by black cohosh is known to contain estrogen responsive elements (ERE) in the promoter region.

### Effects of actein and the cycloartane aglycon mixture

**Figure 9:**
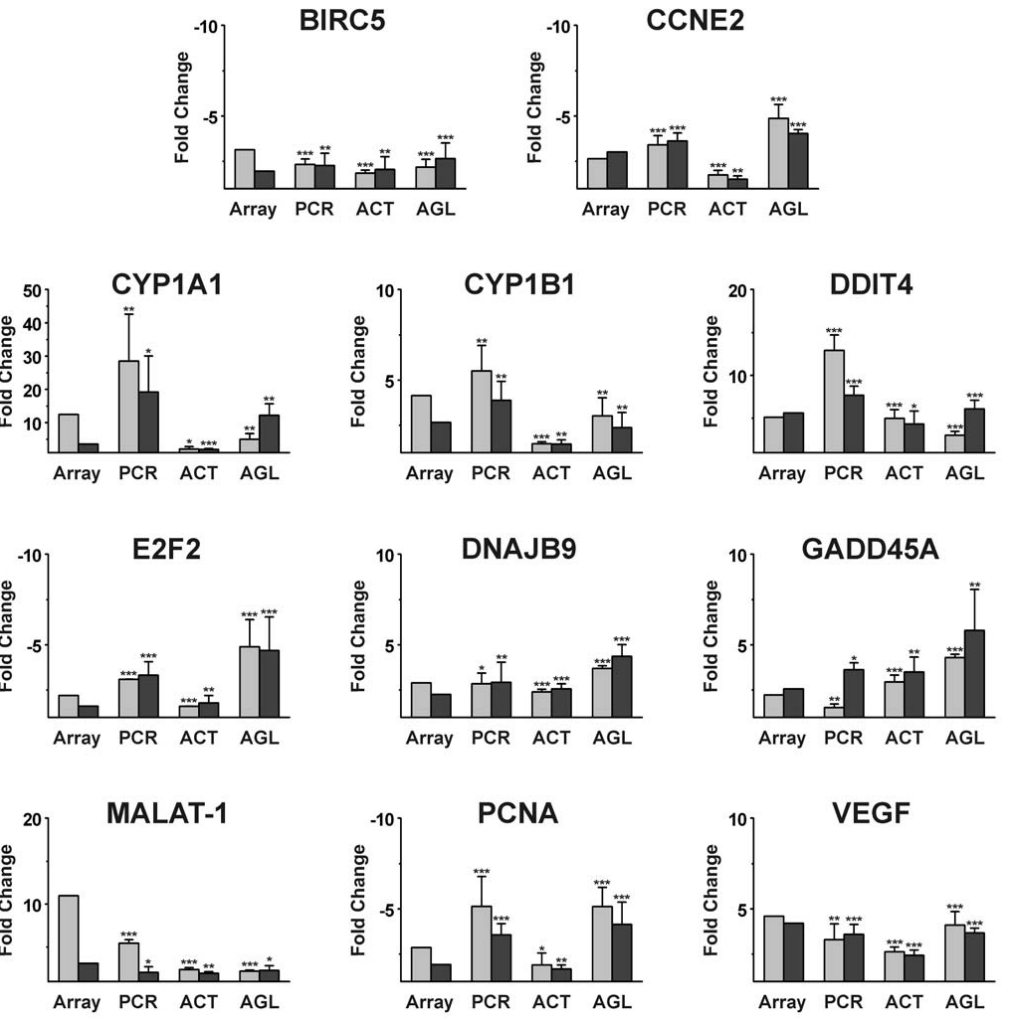
Gene expression levels of selected genes in MCF-7 cells after 24 h treatment with black cohosh extract (15 μg/ml), the triterpene glycoside actein (20 μM) or the triterpene aglycon mixture (30 μM). For extract treatment results obtained with microarrays (**Array**) and real-time RT-PCR (**PCR**) are shown. For actein (**ACT**) and the aglycons (**AGL**) expression levels were determined by real-time RT-PCR. Bars represent gene expression levels as fold changes calculated *versus *DMSO control. RT-PCR measurements were done at least in triplicate. The data are presented as means ± SD (*p < 0.05, **p < 0.01, ***p < 0.001: gene expression statistically significantly different from DMSO control, calculated by Student's t-test).

To identify active principles in black cohosh, MCF-7 cells were treated with the major cycloartane glycoside actein and a mixture of cycloartenol aglycons under the same conditions as in the microarray experiments, at concentrations corresponding to the IC_50 _values determined in the proliferation assay. We used real-time RT-PCR to determine the expression levels *vs*. DMSO control of the 13 genes selected on the basis of the microarray experiments: *baculoviral IAP repeat-containing 5 *(*BIRC5*), *cyclin E2 *(*CCNE2*), *cyclin G2 *(*CCNG2*), *cytochrome P450 1A1 *(*CYP1A1*), *cytochrome P450 1B1 *(*CYP1B1*), *DNA-damage-inducible transcript 4 *(*DDIT4*), *DnaJ (Hsp40) homolog, subfamily B, member 9 *(*DNAJB9*), *E2F transcription factor 2 *(*E2F2*), *estrogen receptor α *(*ESR1*), *growth arrest and DNA-damage-inducible, alpha *(*GADD45A*), *metastasis associated in lung adenocarcinoma transcript 1 *(*MALAT-1*), *proliferating cell nuclear antigen *(*PCNA*) and *vascular endothelial growth factor *(*VEGF*). Upon treatment with actein and the aglycon mixture, all transcripts appeared to be regulated in the same direction and, in a majority of cases, in a comparable order of magnitude as with black cohosh treatment (Figures 8 and 9). In general, treatment with actein resulted in a slightly weaker regulation of the expression levels than treatment with the black cohosh extract. In contrast, the treatment with the aglycon mixture caused a marginally stronger up- or downregulation of some genes, such as *CCNE2*, *DNAJB9*, *E2F2*, *ESR1 *and *GADD45A*. Also the AhR target genes *CYP1A1 *and *CYP1B1 *were upregulated by the aglycon mixture and with actein albeit at a lower level.

## Discussion

We performed the first gene expression profiling experiment with rhizomes of *Cimicifuga racemosa *(black cohosh) to identify molecular effects in the human breast cancer cell line MCF-7. In initial experiments analyzing cell proliferation we observed growth inhibition in response to treatment with a lipophilic extract of black cohosh, the major cycloartane-type triterpene glycoside actein and a cycloartane aglycon mixture. IC_50 _values were comparable to previously reported results [22-24].

Effects of a black cohosh extract on gene expression in MCF-7 cells were determined by means of Affymetrix GeneChip^® ^Human Genome U133 Plus 2.0 arrays, enabling almost complete analysis of the transcriptome. After treatment of MCF-7 cells for 24 h with the black cohosh extract at the IC_50 _concentration (15 μg/ml) changes of the expression levels of 431 genes were detected using highly stringent selection criteria. Random permutation of gene expression profiles showed a mean of 22 to 32 regulated probe sets in our data set with our selection criteria indicating a false positive rate of about 5%.

Comparing the expression patterns, action of black cohosh is opposite to estradiol and more similar to tamoxifen regarding proliferation and cell survival. However, the effect of black cohosh treatment appears more complex, as significantly more genes were regulated than either with E2 or tamoxifen. The genes regulated by black cohosh did not include well-known estrogen-regulated genes, with the exception of *ESR1 *(the estrogen recptor α gene) and *VEGF *which both could be regulated via hypoxia response. In contrast, a wider range of cellular pathways and targets were affected by black cohosh but not E2 or tamoxifen. Hence, action of black cohosh in MCF-7 cells seems to be neither estrogenic nor antiestrogenic but rather multifacetted. Because MCF-7 is a model for determining estrogenic or antiestrogenic effects, we can conclude that the benefical effects of black cohosh in alleviating postmenopausal complaints might be rather due to central nervous action via dopamine, serotonin or *μ *opioidic receptors.

Among all regulated genes, those related to the functional categories of *proliferation *(*cell cycle and DNA replication*) and *stress response *(including members of different functional categories) were found to be highly significantly overrepresented, and these groups appear to be interconnected. Given that many genes were regulated at a lower level and, therefore, excluded by the stringent filter setting, a statistically highly significant accumulation of genes in these functional categories emphasizes the significance of our results.

The cell cycle inhibition observed in the proliferation experiments appears to be due to an arrest at both G1/S- and G2/M-transition points, as indicated by an accumulation of regulated genes. This finding is corroborated by recent results of a flow cytometric analysis [24]. Several genes involved in apoptosis appeared to be regulated, most genes in a pro-apoptotic manner. Some genes are related to apoptotic as well as anti-apoptotic events. These genes are linked to different cellular stress response mechanisms. Altogether, the expression levels of approximatively 40 genes related to stress response were affected by black cohosh. Given the fact that results of stress response pathways are contradictory, pro- and anti-apoptotic gene expression may not be surprising. The main purpose of stress response is an adaptation of the cells to stress factors, resulting in cell survival, angiogenesis and promoted tumor growth. However, when stress reaches a certain threshold, the protecting pathways become saturated and cells undergo apoptosis [49]. This may explain the seemingly contradictory effects observed in our experiment. Altogether, pro-apoptotic signalling seems to outweigh. Stress response regulation has already been previously reported for other xenobiotics such as the cancer chemoprotective phytochemical indole-3-carbinol (I3C) and its physiological condensation product diindolylmethane (DIM), whose antitumor activity has been widely investigated [42]. Its influence on gene expression in different tumor cells – among others in breast cancer cells such as MCF-7 – shows a certain similarity to our results with black cohosh. However, the mechanism of induced stress response by I3C is still unknown.

In association with unfolded protein response various transcripts of protein turnover were affected by black cohosh. Regulation of several transcripts whose products are involved in ubiquitinylation may be due to augmented degradation of malformed proteins. Increased mRNA levels of not less than eight different aminoacyl-tRNA synthetases (ARSs) are not only linked to protein synthesis. Some ARSs are rather multifunctional proteins involved in different cellular processes [61]. For example, the secretion of tyrosyl-tRNA synthetase (YARS) is linked to apoptotic events [39] and tryptophanyl-tRNA synthetase (WARS) has been shown to possess angiostatic and proliferation-inhibitory activity [61].

As response to exposure to black cohosh extract a group of transcripts coding for enzymes with oxidoreductase activity was upregulated ("response to xenobiotics"). A strong upregulation was observed for *CYP1A1*, and, to a somewhat lesser extent, of *CYP1B1*. Apart from well-known involvement in xenobiotic metabolism, thereby mediating toxic and tumorigenic effects of several chemicals, these two oxidoreductases are involved in the metabolism of 17β-estradiol [62]. CYP1A1 metabolizes E2 to non-carcinogenic 2-hydroxy-E2 whereas CYP1B1 is responsible for the formation of carcinogenic 4-hydroxy-E2. The two enzymes are not always expressed at the same level in tissues. An increased production of 2-hydroxy-E2 relative to 4-hydroxy-E2, due to a higher expression level of CYP1A1 than CYP1B1, has been suggested to be contributing to the antitumor activity of indol-3-carbinol and, therefore, being of clinical importance [62]. As described, we also observed significantly stronger induction of CYP1A1 transcripts than CYP1B1 with black cohosh treatment.

*CYP1A1 *is known as *the *classical target of the aryl hydrocarbon receptor (AhR). Interestingly, the receptor has also been upregulated with our experiment. The AhR, upon binding of a ligand, forms a heterodimeric complex with ARNT (aryl hydrocarbon receptor nuclear translocator) which induces transactivation of the CYPs and other target genes via binding to xenobiotic response elements (XREs) in their promoter regions [63]. Classical AhR ligands and, therefore, CYP1A1 inducers are hydrophobic and planar or coplanar molecules of polycyclic structure. Although the cycloartane-type triterpenoids do not satisfy these structural requirements, gene expression of *CYP1A1 *was not only induced in our experiments by black cohosh extract but also by the cycloartane aglycons (5.0 ± 1.7 fold, p < 0.01 and 12.2 ± 3.4 fold, p < 0.01) and, to a lower but statistically significant extent, by actein (2.1 ± 0.8, p < 0.05 and 2.0 ± 0.2, p < 0.01). *CYP1B1 *was also differentially expressed by the triterpenoids. The upregulation of *CYP1A1 *and *CYP1B1 *expression by extract and purified cycloartanes cannot be explained by AhR binding. Indeed, black cohosh extract and compounds did not show any AhR activity in a reporter gene assay [64] in rat hepatoma H4IIE cells [see Additional file 2]. These findings are in contradiction with previous experiments showing weak AhR binding and transactivation activity of black cohosh extracts. However, these experiments were carried out with high concentrations (up to 200 μg/ml) and did not include single compounds [20,21]. The upregulation of *CYP1A1 *and *CYP1B1 *may be linked to activation of genes involved with hypoxic stress response, although the consequences of upregulated stress response appear somewhat contradictory. AhR and hypoxia-induced signal transduction are connected via ARNT, and AhR and HIF1α compete in binding to ARNT. Hypoxic conditions reportedly inhibited AhR-dependent gene expression [65], on the other hand, a connection between *CYP1A1 *induction and hypoxia-induced gene regulation in Caco2 cells has been found [66].

Besides *CYP1A1 *and *CYP1B1*, 11 other selected mRNAs representing all regulated functional categories were investigated by real-time RT-PCR to verify the microarray results and to investigate the effects of the cycloartane-type triterpenoids. Briefly, all genes were regulated by the triterpenoids in the same direction and to a fairly comparable extent than with the extract. Hence, we provide here first evidence that cycloartane glycosides as well as their aglycons, which are most likely formed in the intestine prior to absorption, are putative active principles in black cohosh.

## Conclusion

In conclusion, the microarray investigation on black cohosh in MCF-7 breast cancer cells revealed several new interesting aspects. Gene expression "contra cell proliferation" and "pro apoptosis" emphasizes previously reported actions of black cohosh. These effects might be a result of stress response to hypoxic conditions, protein disturbances and induction of oxidoreductases, evoked by an exposure to black cohosh extract. Regulation of other genes, eg. induction of *CYP1A1*, *IFNGR1 *and *WARS *and downregulation of ERα mRNA (*ESR1*) might contribute to a putative antitumor activity of black cohosh. On the other hand, activation of genes involved in cell survival, angiogenesis and tumor progression, such as *VEGF*, *S100P*, *MALAT-1 *and different stress response genes, raises questions. Since black cohosh is increasingly popular as an alternative to HRT, a thorough benefit/risk assessment is required. Black cohosh preparations have been considered as safe up to now and no serious adverse reactions have been reported so far. Nonetheless, further investigations are needed to assess the safety level and to analyze putative antitumor applications with functional assays and *in vivo *models.

## Competing interests

The author(s) declare that there are no competing interests.

## Authors' contributions

MH and SW conceived the study and supervised its performance. FG carried out all experiments with the exception of RNA isolation and the following steps of the microarray experiment which were performed by LP. Data analysis was performed by TK, FG and SW. FG and SW drafted the manuscript and MH participated in its preparation. All authors have read and approved the manuscript.

## Supplementary Material

Additional file 1 Differentially expressed genes. The table lists all 431 genes differentially expressed (>1.5 fold in two parallel experiments) by black cohosh extract in MCF-7 cells. Click here for file

Additional file 2 Aryl hydrocarbon receptor activity. The diagram presents the results of a XRE-dependent reporter gene assay in rat H4IIE cells. Click here for file
