# Supplementary material for: Gene expression profiling reveals effects of Cimicifuga racemosa (L.) NUTT. (black cohosh) on the estrogen receptor positive human breast cancer cell line MCF-7
Source: BMC Pharmacol. 2007 Sep 20;7:11. doi: 10.1186/1471-2210-7-11 (PMC2194763; doi:10.1186/1471-2210-7-11)
Supplement: Additional file 1 — Differentially expressed genes. The table lists all 431 genes differentially expressed (>1.5 fold in two parallel experiments) by black cohosh extract in MCF-7 cells. [file 1471-2210-7-11-S1.pdf]

ADDITIONAL FILE 1

Gene expression profiling reveals effects of *Cimicifuga racemosa* (L.) NUTT. (black cohosh) on the estrogen receptor positive human breast cancer cell line MCF-7

Friedemann Gaube, Stefan Wölfl, Larissa Pusch, Torsten C Kroll and Matthias Hamburger

List of all 431 genes differentially expressed by black cohosh extract in MCF-7 cells. Genes are listed with symbol, GeneBank accession number and fold changes vs. DMSO control of two independent microarray experiments.

Gene Symbol	Gene Title	Accession No.	Fold Change # 1	# 2
---	CDNA FLJ42435 fis, clone BLADE2006849	AA042983	3.2	2.3
---	Transcribed sequence with moderate similarity to protein sp:P39193 (H.sapiens) ALU6_HUMAN Alu subfamily SP sequence contamination warning entry	AA740632	1.7	1.8
---	gastric-associated differentially-expressed protein YA61P, drug sensitive protein 1	AF220415	2.1	5.6
---	M41 mRNA, complete sequence; alternatively spliced	AF401033	1.6	1.6
---	Clone IMAGE:5261280, mRNA	AI015847	1.9	1.7
---	CDNA FLJ11381 fis, clone HEMBA1000501	AI889959	-1.9	-2.1
---	C20orf119, CDNA FLJ42053 fis, clone SPLEN2042535, moderately similar to POLYADENYLATE-BINDING PROTEIN 1	AL109839	2.8	3.4
---	CDNA FLJ46882 fis, clone UTERU3015844	AV709727	1.7	2.0
---	Uncharacterized gastric protein ZA31P	AW256031	1.6	1.6
---	CDNA clone IMAGE:5286843, partial cds	AW952781	2.2	1.9
---	Clone IMAGE:3881549, mRNA	BE222344	1.8	3.1
---	ACA24 snoRNA gene; Clone IMAGE:4249217, mRNA	BF340290	1.9	1.9
AACS	acetoacetyl-CoA synthetase	NM_023928	1.6	1.5
ABAT	4-aminobutyrate aminotransferase	AF237813	1.5	1.6
ABCA12	ATP-binding cassette, sub-family A (ABC1), member 12	AL080207	1.5	2.5
ABCG1	ATP-binding cassette, sub-family G (WHITE), member 1	NM_004915	1.7	1.6
ACSL1	acyl-CoA synthetase long-chain family member 1	NM_021122	2.2	2.6
ACSL3	acyl-CoA synthetase long-chain family member 3	NM_004457	1.8	1.7
AF15Q14 (D40)	AF15q14 protein	BF248364	-1.6	-1.5
AFTIPHILIN	aftiphilin protein	BF055271	1.6	1.6
AGA	aspartylglucosaminidase	NM_000027	1.6	2.4
AHR	aryl hydrocarbon receptor	NM_001621	1.8	1.8
AIG1	androgen-induced 1	AF151861	1.7	1.6
AKR1C1	aldo-keto reductase family 1, member C1 (dihydrodiol dehydrogenase 1; 20-alpha (3-alpha)-hydroxysteroid dehydrogenase)	M33376	2.3	5.5
ALAS1	aminolevulinate, delta-, synthase 1	NM_000688	1.7	2.0
ALCAM	activated leukocyte cell adhesion molecule	BF242905	1.6	2.0
ALDH1L2	aldehyde dehydrogenase 1 family, member L2	AI654224	2.2	5.0
ALDH3B2	aldehyde dehydrogenase 3 family, member B2	AA071510	2.1	2.3
AMIGO2	amphoterin induced gene 2	AC004010	1.6	1.9
ANK3	ankyrin 3, node of Ranvier (ankyrin G)	NM_020987	1.6	1.6
ANKRD11	ankyrin repeat domain 11	BE890185	1.5	2.1
ANLN	anillin, actin binding protein (scraps homolog, Drosophila)	NM_018685	-1.9	-2.3
APG12L (ATG12)	APG12 autophagy 12-like (S. cerevisiae)	BE965998	1.7	1.6
ARFGEF2 (SNAP23P)	ADP-ribosylation factor guanine nucleotide-exchange factor 2 (brefeldin A-inhibited) ; synaptosomal-associated protein, 23kDa pseudogene	AL121903	1.8	1.8
ARHE	ras homolog gene family, member E	BG054844	1.6	1.6
ARHGDIA	Rho GDP dissociation inhibitor (GDI) alpha	AI571798	-2.0	-1.9
ARMCX3	armadillo repeat containing, X-linked 3	NM_016607	1.9	2.1
ARMCX5 (FLJ12969)	armadillo repeat containing, X-linked 5	NM_022838	1.6	1.5
ARNTL	aryl hydrocarbon receptor nuclear translocator-like	AB000815	1.8	1.6
ARPC4	actin related protein 2/3 complex, subunit 4, 20kDa	AF019888	-1.6	-1.6
ASF1B	ASF1 anti-silencing function 1 homolog B (S. cerevisiae)	NM_018154	-2.1	-3.5
ASNS	asparagine synthetase	NM_001673	4.1	3.7
ASPH	aspartate beta-hydroxylase	AF289489	2.2	2.4
ASS	argininosuccinate synthetase	NM_000050	1.6	1.8
ATAD2	ATPase family, AAA domain containing 2	NM_014109	-1.7	-2.6
ATF3	activating transcription factor 3	NM_001674	2.0	1.8

Gene Symbol	Gene Title	Accession No.	Fold Change # 1	# 2
ATF4 (CREB2)	activating transcription factor 4 (tax-responsive enhancer element B67)	NM_001675	1.5	1.7
ATXN1 (SCA1)	spinocerebellar ataxia 1 (olivopontocerebellar ataxia 1, autosomal dominant, ataxin 1)	NM_000332	1.5	3.2
B3GNT5	UDP-GlcNAc:betaGal beta-1,3-N-acetylglucosaminyltransferase 5	BE672260	1.7	2.0
BEX2	brain expressed X-linked 2	AF251053	3.6	4.6
BHLHB2 (DEC1, SHARP2)	basic helix-loop-helix domain containing, class B, 2	NM_003670	2.6	3.6
BIRC4	baculoviral IAP repeat-containing 4	BF109251	1.5	1.8
BIRC5	baculoviral IAP repeat-containing 5 (survivin)	AA648913	-2.0	-3.1
BNIP3L	BCL2/adenovirus E1B 19kDa interacting protein 3-like	AL132665	1.6	1.8
BRIP1	BRCA1 interacting protein C-terminal helicase 1	AF360549	-1.7	-2.6
BRRN1	barren homolog (Drosophila)	D38553	-1.6	-2.0
C10orf18	chromosome 10 open reading frame 18	NM_017782	1.5	1.8
C12orf22	chromosome 12 open reading frame 22	NM_030809	1.8	1.8
C14orf101	chromosome 14 open reading frame 101	NM_017799	1.9	1.8
C14orf147	chromosome 14 open reading frame 147	BE738425	1.8	2.2
C15orf15	chromosome 15 open reading frame 15	AF165521	1.9	1.8
C1orf19	chromosome 1 open reading frame 19	AF288394	2.2	1.9
C1orf22	chromosome 1 open reading frame 22	BF439488	1.8	1.6
C1orf24	chromosome 1 open reading frame 24	AF288391	5.1	4.9
C1orf34	chromosome 1 open reading frame 34	BC004399	1.8	1.5
C20orf129	chromosome 20 open reading frame 129	BC001068	-1.5	-2.2
C20orf27	chromosome 20 open reading frame 27	AI761506	-1.5	-1.7
C20orf36	chromosome 20 open reading frame 36	AB028973	1.6	1.6
C6orf211	chromosome 6 open reading frame 211	NM_024573	-1.5	-1.6
C9orf152	chromosome 9 open reading frame 152	AI380443	1.7	1.7
C9orf64	chromosome 9 open reading frame 64	AW983691	1.6	1.8
CAPN2	calpain 2, (m/II) large subunit	M23254	1.5	1.5
CARS	cysteinyl-tRNA synthetase	AI769685	2.0	1.9
CBS	cystathionine-beta-synthase	BE613178	2.0	2.0
CBX4	chromobox homolog 4 (Pc class homolog, Drosophila)	AI570531	1.8	1.5
CBX5	Chromobox homolog 5 (HP1 alpha homolog, Drosophila); Heterochromatin protein-1 (HP1); Clone IMAGE:5288883, mRNA	AA181060	-1.7	-2.5
CCAR1 (CARP1)	cell division cycle and apoptosis regulator 1	BE617899	1.6	1.6
CCNA2	cyclin A2	NM_001237	-1.5	-2.3
CCNB1IP1	cyclin B1 interacting protein 1	NM_021178	1.6	1.6
CCNE2	cyclin E2	AF112857	-3.0	-2.6
CCNF (FBXO1; FBX1)	cyclin F	U17105	-1.9	-1.5
CCNG2	cyclin G2	AW134535	2.3	2.8
CDC6	CDC6 cell division cycle 6 homolog (S. cerevisiae)	NM_001254	-1.6	-2.5
CDK2	cyclin-dependent kinase 2	AB012305	-1.5	-2.1
CDK7	cyclin-dependent kinase 7 (MO15 homolog, Xenopus laevis, cdk-activating kinase)	L20320	1.6	1.6
CDKN1A	cyclin-dependent kinase inhibitor 1A (p21, Cip1)	NM_000389	1.6	1.8
CDKN2C	cyclin-dependent kinase inhibitor 2C (p18, inhibits CDK4)	NM_001262	-1.7	-2.0
CDT1	DNA replication factor	AF321125	-1.7	-2.5
CDYL	chromodomain protein, Y-like	AL050164	1.8	1.7
CEBPB	CCAAT/enhancer binding protein (C/EBP), beta	AL564683	2.3	2.4
CEBPG	CCAAT/enhancer binding protein (C/EBP), gamma	NM_001806	2.4	2.2
CGI-85 (SUV420H1)	CGI-85 protein; suppressor of variegation 4-20 homolog 1 (Drosophila)	NM_017635	1.5	1.8
CHAF1A	chromatin assembly factor 1, subunit A (p150)	BF062223	-1.6	-1.7
CHMP5	chromatin modifying protein 5	NM_015961	1.5	2.0
CITED2	Cbp/p300-interacting transactivator, with Glu/Asp-rich carboxy-terminal domain, 2	AF109161	1.5	3.1
CLCN3	chloride channel 3	NM_001829	1.6	2.1
CLIC4	chloride intracellular channel 4	NM_013943	2.0	1.8
CLTC	Clathrin, heavy polypeptide (Hc)	AA747756	1.8	1.9
COG6	component of oligomeric golgi complex 6	AF116827	1.5	1.7
COH1 (VPS13B)	Cohen syndrome 1; vacuolar protein sorting 13B (yeast)	AI052003	1.5	1.7
COL1A1	collagen, type I, alpha 1	BE221212	1.6	2.9
CSNK1G3	casein kinase 1, gamma 3	NM_004384	1.6	1.5
CYP1A1	cytochrome P450, family 1, subfamily A, polypeptide 1	NM_000499	3.5	12.4
CYP1B1	cytochrome P450, family 1, subfamily B, polypeptide 1	NM_000104	2.7	4.1
CYP51A1	cytochrome P450, family 51, subfamily A, polypeptide 1 (Sterol 14-alpha-demethylase)	NM_000786	1.6	1.6
D2LIC	dynein 2 light intermediate chain	NM_016008	1.6	1.8
DAF	decay accelerating factor for complement (CD55, Cromer blood group system)	NM_000574	3.3	3.6

Gene Symbol	Gene Title	Accession No.	Fold Change # 1	# 2
DCLRE1B	DNA cross-link repair 1B (PSO2 homolog, S. cerevisiae)	AI703304	-1.7	-2.3
DDIT3 (GADD153, CHOP)	DNA-damage-inducible transcript 3	BC003637	2.7	2.6
DDIT4 (REDD1, RTP801)	DNA-damage-inducible transcript 4	NM_019058	5.6	5.1
DDX11	DEAD/H (Asp-Glu-Ala-Asp/His) box polypeptide 11 (CHL1-like helicase homolog, S. cerevisiae)	AI983033	-1.6	-1.6
DEPDC6	DEP domain containing 6	NM_022783	2.5	2.8
DERP12	DERP12 (dermal papilla derived protein 12)	AB014766	1.5	1.7
DHX9	DEAH (Asp-Glu-Ala-His) box polypeptide 9	BF313832	-1.7	-1.6
DIPA	hepatitis delta antigen-interacting protein A	NM_006848	-1.6	-1.6
DKFZP564I1171	DKFZP564I1171 protein	BF528646	1.5	1.5
DLX1	distal-less homeo box 1	BF060783	2.4	2.4
DNAJB9	DnaJ (Hsp40) homolog, subfamily B, member 9	AL080081	2.3	2.9
DNAJC10	DnaJ (Hsp40) homolog, subfamily C, member 10	BG168666	1.9	3.1
DOCK5	dedicator of cytokinesis 5	BF447954	1.7	2.0
DPYSL2	dihydropyrimidinase-like 2	NM_001386	1.6	1.9
DSIPI	delta sleep inducing peptide, immunoreactor	AL110191	2.0	1.8
DUSP16	dual specificity phosphatase 16	AB052156	1.5	1.6
DUSP4	dual specificity phosphatase 4	NM_001394	2.1	2.8
E2F2	E2F transcription factor 2	AL561296	-1.6	-2.2
E2F7	E2F transcription factor 7	AI341146	-2.1	-2.7
EFEMP1	EGF-containing fibulin-like extracellular matrix protein 1	AI826799	-1.7	-2.4
EGR1	early growth response 1	AI459194	4.7	1.7
EHF (ESE3)	ets homologous factor (ESE3)	AI763378	1.8	2.0
EIF1 (SUI1)	putative translation initiation factor (eucaryotic translation initiation factor 1)	AF083441	1.7	1.7
EIF2AK3 (PERK)	eukaryotic translation initiation factor 2-alpha kinase 3	NM_004836	1.7	2.2
EIF4EBP1	eukaryotic translation initiation factor 4E binding protein 1	AB044548	1.8	2.3
ELF5	E74-like factor 5 (ets domain transcription factor)	AF115403	1.6	1.6
ENPP1	ectonucleotide pyrophosphatase/phosphodiesterase 1	NM_006208	1.7	1.7
EPAS1 (HIF2a)	endothelial PAS domain protein 1	AF052094	1.9	2.1
EPRS	glutamyl-prolyl-tRNA synthetase	NM_004446	1.7	1.8
ESR1	estrogen receptor 1	NM_000125	-1.8	-2.0
EVL	Enah/Vasp-like	NM_016337	-1.6	-1.7
F7	coagulation factor VII (serum prothrombin conversion accelerator)	NM_000131	1.8	2.1
FAM18B	family with sequence similarity 18, member B	NM_016078	1.6	1.9
FBXO11	F-box only protein 11	AL117620	1.7	1.9
FBXO5	F-box only protein 5 (early mitotic inhibitor 1)	AK026197	-1.7	-2.0
FDFT1	farnesyl-diphosphate farnesyltransferase 1 (squalene synthase)	AA872727	1.7	1.6
FEN1	flap structure-specific endonuclease 1	NM_004111	-2.5	-3.4
FIGNL1	fidgetin-like 1	AK023411	-1.9	-2.0
FLJ10330	sarcoma antigen NY-SAR-27; hypothetical protein FLJ10330	N32872	1.6	1.8
FLJ10618	hypothetical protein FLJ10618	AI927944	1.9	2.3
FLJ10719	hypothetical protein FLJ10719	BC004277	-1.7	-2.4
FLJ10980	hypothetical protein FLJ10980	AB037791	1.7	2.1
FLJ11273	hypothetical protein FLJ11273	AV705186	1.6	1.8
FLJ13448	hypothetical protein FLJ13448	NM_025147	1.6	1.7
FLJ14007	hypothetical protein FLJ14007	NM_024699	1.8	2.6
FLJ20160	FLJ20160 protein	AA133311	2.0	1.7
FLJ21439	hypothetical protein FLJ21439	NM_025137	1.6	2.1
FLJ21657	hypothetical protein FLJ21657	NM_022483	1.5	2.2
FLJ22104	hypothetical protein FLJ22104	AK000684	1.8	1.6
FLJ23053	hypothetical protein FLJ23053	NM_022907	1.5	1.6
FLJ30428	CDNA FLJ30428 fis, clone BRACE2008941	AW188087	1.5	2.2
FLJ31951	hypothetical protein FLJ31951	AL553942	1.8	3.5
FLJ39370	hypothetical protein FLJ39370	AI110850	1.7	1.8
FNDC3	fibronectin type III domain containing 3	NM_014923	1.5	2.1
FOLR1	folate receptor 1 (adult)	AF000381	1.6	2.1
FOXO3A	forkhead box O3A	AV725666	1.9	1.8
FOXP1	forkhead box P1	AK026898	1.7	1.7
FST	follistatin	BF438173	2.2	3.3
FTH1	ferritin, heavy polypeptide 1	AA083483	1.9	1.9
GABARAPL1	GABA(A) receptor-associated protein like 1	AF180519	2.1	1.7
GADD45A	growth arrest and DNA-damage-inducible, alpha	NM_001924	2.6	2.2
GALNT7	UDP-N-acetyl-alpha-D-galactosamine:polypeptide N-acetylgalactosaminyltransferase 7 (GalNAc-T7)	NM_017423	1.8	1.9
GARNL1	GTPase activating RANGAP domain-like 1	BG436400	1.8	2.1
GARS	glycyl-tRNA synthetase	D30658	2.3	1.7
GDF15	growth differentiation factor 15; prostate derived-factor (PDF)	AF003934	4.5	6.0

Gene Symbol	Gene Title	Accession No.	Fold Change # 1	# 2
GFPT1	glutamine-fructose-6-phosphate transaminase 1	AI268315	1.9	3.2
GLRX	glutaredoxin (thioltransferase)	AF162769	1.9	3.0
GMNN	geminin, DNA replication inhibitor	NM_015895	-1.7	-2.2
GOLGA5	golgi autoantigen, golgin subfamily a, 5	NM_005113	1.5	1.7
GOT1	glutamic-oxaloacetic transaminase 1, soluble (aspartate aminotransferase 1)	BC000498	1.7	1.6
GPR56	G protein-coupled receptor 56	AL554008	1.6	1.6
GPT2	glutamic pyruvate transaminase (alanine aminotransferase) 2	BG328998	2.3	1.7
GTPBP2	GTP binding protein 2	NM_019096	2.1	2.1
H2AFX	H2A histone family, member X	AA760862	-1.8	-2.6
hCAP-D3 (KIAA0056)	KIAA0056 protein	AI796581	-1.6	-1.6
HELLS; PASG	helicase, lymphoid-specific	AI650364	-2.7	-2.8
HERPUD1	homocysteine-inducible, endoplasmic reticulum stress-inducible, ubiquitin-like domain member 1	AF217990	2.0	2.5
HIF1A	hypoxia-inducible factor 1, alpha subunit (basic helix-loop-helix transcription factor)	NM_001530	1.5	1.5
HIST1H2AC	histone 1, H2ac	AL353759	1.5	1.9
HIST2H2BE	histone 2, H2be, Histone H2B.q	NM_003528	2.1	2.3
HMGCR	3-hydroxy-3-methylglutaryl-Coenzyme A reductase	AL518627	1.7	1.8
HMGCS1	3-hydroxy-3-methylglutaryl-Coenzyme A synthase 1 (soluble)	BG035985	1.6	2.2
HMOX1	heme oxygenase (decycling) 1	NM_002133	2.1	3.3
HNRPDL (JKTBP2)	heterogeneous nuclear ribonucleoprotein D-like; JKTBP2 (hnRNP JKTBP) (A+U-rich element RNA binding factor)	AB066484	-1.7	-1.6
HNRPUL1 (E1BAP5)	heterogeneous nuclear ribonucleoprotein U-like 1	BC004242	-1.5	-1.8
HS6ST2	heparan sulfate 6-O-sulfotransferase 2	NM_147174	1.5	1.8
HSPA1A (HSPA1B)	heat shock 70kDa protein 1A	NM_005345	-1.5	-1.5
HSPA2	heat shock 70kDa protein 2	U56725	-1.9	-1.7
HSPA5	heat shock 70kDa protein 5 (glucose-regulated protein, 78kDa)	AF216292	1.6	1.8
HTPAP	HTPAP protein	BE858787	1.7	2.1
IARS	isoleucine-tRNA synthetase	NM_013417	2.0	1.9
IDH1	isocitrate dehydrogenase 1 (NADP+), soluble	BC012846	1.8	2.3
IDS	iduronate 2-sulfatase (Hunter syndrome)	NM_000202	1.6	1.6
IER3 (IEX1)	immediate early response 3	NM_003897	1.9	2.0
IFNGR1	interferon gamma receptor 1	NM_000416	1.6	2.1
IFRD1	interferon-related developmental regulator 1	AA747426	2.9	3.7
INPP4B	inositol polyphosphate-4-phosphatase, type II, 105kDa	NM_003866	1.5	1.5
INSIG1	insulin induced gene 1	BG292233	1.8	2.4
INVS	inversin	AF039217	1.7	1.6
IPLA2 (GAMMA)	intracellular membrane-associated calcium-independent phospholipase A2 gamma	AF217519	1.9	1.8
IRF2BP2	interferon regulatory factor 2 binding protein 2	AW242432	1.8	2.9
IRS1	insulin receptor substrate 1	NM_005544	1.5	1.6
IRX3	iroquois homeobox protein 3	AI681917	1.9	2.3
ISG20	interferon stimulated gene 20kDa	NM_002201	1.5	1.6
JARID1B (RBBP2H1A; PLU-1)	Jumonji, AT rich interactive domain 1B (RBP2-like), retinoblasomta-binding protein 2, homolog 1A (RBBP2H1A)	NM_006618	1.6	1.9
JMY	junction-mediating and regulatory protein	BF447037	1.6	2.2
JUB	jub, ajuba homolog (Xenopus laevis)	AI289311	1.8	1.6
KCNE4	potassium voltage-gated channel, Isk-related family, member 4	AI002715	2.1	3.1
KDELR2	KDEL (Lys-Asp-Glu-Leu) endoplasmic reticulum protein retention receptor 2	BG026159	1.5	1.7
KIAA0323	KIAA0323	AI075450	1.5	1.6
KIAA0436	putative prolyl oligopeptidase	AB007896	1.7	1.6
KIAA0703	Probable calcium-transporting ATPase KIAA0703 (EC 3.6.3.8)	NM_014861	1.6	1.6
KIAA0746	KIAA0746 protein	AB018289	1.5	1.9
KIAA0776	KIAA0776	AW298092	1.6	1.5
KIAA0888	KIAA0888 protein	AV720650	1.6	1.6
KIAA1102	KIAA1102 protein	AK027231	1.7	2.0
KIAA1946	KIAA1946	AW043602	1.5	1.7
KIF11	kinesin family member 11 (Eg5)	NM_004523	-1.7	-2.7
KLF4	Kruppel-like factor 4 (gut)	BF514079	1.8	1.5
KMO	kynurenine 3-monooxygenase (kynurenine 3-hydroxylase)	AI074145	2.6	2.8
LACTB2	lactamase, beta 2	BC000878	1.6	1.7
LDLR	low density lipoprotein receptor (familial hypercholesterolemia)	NM_000527	1.8	1.8
LMNB1	lamin B1	NM_005573	-1.7	-2.1
LOC144871	hypothetical protein LOC144871	BG913589	1.5	1.8
LOC145741	hypothetical LOC145741	BE218239	1.8	3.2

Gene Symbol	Gene Title	Accession No.	Fold Change # 1	# 2
LOC149603	hypothetical protein LOC149603	AA085748	1.8	1.6
LOC222171	hypothetical protein LOC222171	AI347918	3.6	5.1
LOC286144	hypothetical protein LOC286144	AW303300	1.5	1.7
LOC51136	PTD016 protein	N51514	1.5	2.0
LOC51249 (C1orf154)	hypothetical protein LOC51249; chromosome 1 open reading frame 154	AF151063	1.5	1.5
LOC51315	hypothetical protein LOC51315	NM_016618	1.6	1.8
LPIN1	lipin 1	D80010	1.6	2.4
LXN	latexin	NM_020169	2.1	1.9
MALAT-1	metastasis associated in lung adenocarcinoma transcript 1	BG534952	3.1	11.0
MAP1LC3B	microtubule-associated protein 1 light chain 3 beta	AF183417	1.9	2.2
MAPK6 (ERK3)	mitogen-activated protein kinase 6	NM_002748	1.5	1.5
MAPK8 (JNK1)	mitogen-activated protein kinase 8; (Stress-activated protein kinase JNK1) (c-Jun N-terminal kinase 1) (JNK-46).	AU152505	1.5	1.5
MARS	methionine-tRNA synthetase	AA621558	2.2	2.2
MCM10	MCM10 minichromosome maintenance deficient 10 (S. cerevisiae)	NM_018518	-1.8	-3.8
MCM2	MCM2 minichromosome maintenance deficient 2, mitotin (S. cerevisiae)	NM_004526	-1.6	-3.1
MCM3	MCM3 minichromosome maintenance deficient 3 (S. cerevisiae)	NM_002388	-1.8	-2.1
MCM4	MCM4 minichromosome maintenance deficient 4 (S. cerevisiae)	AI859865	-2.4	-3.4
MCM5	MCM5 minichromosome maintenance deficient 5, cell division cycle 46 (S. cerevisiae)	AA807529	-1.7	-2.6
MCM7	MCM7 minichromosome maintenance deficient 7 (S. cerevisiae)	AF279900	-1.8	-2.7
MDM4	Mdm4, transformed 3T3 cell double minute 4, Mdm2-like p53 binding protein (mouse)	AA745971	1.5	1.5
ME1	malic enzyme 1, NADP(+)-dependent, cytosolic	NM_002395	1.8	2.2
MGC11324	hypothetical protein MGC11324	BC006236	1.8	1.8
MGC21518	hypothetical protein MGC21518	AW054855	1.5	1.5
MGC24665	hypothetical protein MGC24665	AW138157	-1.5	-2.1
MGC34646	hypothetical protein MGC34646	N30209	2.2	3.0
MGC4504	hypothetical protein MGC4504	NM_024111	3.3	2.1
MGC5576	hypothetical protein MGC5576	NM_024056	-1.6	-1.9
MIG-6	mitogen-inducible gene 6	AL034417	2.5	2.1
MINA	MYC induced nuclear antigen	AI823896	1.6	1.9
MKI67	antigen identified by monoclonal antibody Ki-67, proliferation-related Ki-67 antigen	AU152107	-1.9	-2.3
MKNK2	MAP kinase-interacting serine/threonine kinase 2	NM_017572	2.0	2.4
MLPH	Melanophilin	AI810764	1.6	2.0
MOCOS	molybdenum cofactor sulfurase	NM_017947	2.1	2.2
MOSPD1	motile sperm domain containing 1	NM_019556	1.7	1.8
MSH6	mutS homolog 6 (E. coli)	D89646	-1.6	-1.7
MTAC2D1	membrane targeting (tandem) C2 domain containing 1	NM_152332	1.5	2.2
MTHFD2	methylene tetrahydrofolate dehydrogenase (NAD+ dependent), methenyltetrahydrofolate cyclohydrolase	NM_006636	2.0	1.9
MTRR	5-methyltetrahydrofolate-homocysteine methyltransferase reductase	NM_024010	2.2	2.3
MUC5B	mucin 5, subtype B, tracheobronchial	AI697108	1.5	1.7
MXI1	MAX interactor 1	NM_005962	1.7	1.8
MYOZ2	myozenin 2 (calcisarcin 1)	AI475544	3.8	4.5
NCOA7	nuclear receptor coactivator 7	AL035689	1.6	1.7
NFE2L1	nuclear factor (erythroid-derived 2)-like 1	NM_003204	1.6	1.6
NFE2L2	nuclear factor (erythroid-derived 2)-like 2	NM_006164	1.5	2.0
NTN4	netrin 4	AF278532	1.6	1.6
NUCB2	nucleobindin 2	NM_005013	1.5	1.6
NUSAP1	nucleolar and spindle associated protein 1	NM_018454	-1.7	-2.0
OCIAD1(OCIA)	OCIA domain containing 1 (ovarian carcinoma immunoreactive antigen)	AF323665	1.6	1.5
OLFM1	olfactomedin 1; Noelin precursor (Neuronal olfactomedin-related ER localized protein)	R38389	-1.5	-1.5
P8	p8 protein (candidate of metastasis 1)	AF135266	2.7	4.4
PANK3	pantothenate kinase 3	AL565516	1.7	1.8
PCK2	phosphoenolpyruvate carboxykinase 2 (mitochondrial)	NM_004563	3.3	2.5
PCNA	proliferating cell nuclear antigen	NM_002592	-1.9	-2.9
PDE8A	phosphodiesterase 8A	W73272	1.5	1.9
PEG10	paternally expressed 10	BE858180	-1.7	-1.5
Pfs2	DNA replication complex GINS protein PSF2	BC003186	-1.5	-3.3
PHGDH	phosphoglycerate dehydrogenase	NM_006623	2.2	2.0
PIGF	phosphatidylinositol glycan, class F	BE897886	2.0	1.8
PIK3R3	phosphoinositide-3-kinase, regulatory subunit, polypeptide 3 (p55, gamma)	BE622627	1.7	2.2
PIR	Pirin	NM_003662	1.5	1.6
PKMYT1	membrane-associated tyrosine- and threonine-specific cdc2-inhibitory kinase	NM_004203	-1.6	-1.8
PLCB1	phospholipase C, beta 1 (phosphoinositide-specific)	AL049593	2.0	2.5

Gene Symbol	Gene Title	Accession No.	Fold Change # 1	# 2
PMAIP1 (NOXA)	phorbol-12-myristate-13-acetate-induced protein 1	AI857639	1.6	1.9
PMP22 (GAS3)	peripheral myelin protein 22, growth arrest specific 3	L03203	-1.9	-2.6
POLA	polymerase (DNA directed), alpha	NM_016937	-1.5	-1.5
POLE3	polymerase (DNA directed), epsilon 3 (p17 subunit)	BC004170	-1.5	-1.9
PPM1E	protein phosphatase 1E (PP2C domain containing)	NM_014906	1.9	1.8
PRNP	prion protein (p27-30) (Creutzfeld-Jakob disease, Gerstmann-Strausler-Scheinker syndrome, fatal familial insomnia)	NM_000311	1.9	2.3
PRSS15	protease, serine, 15; Lon protease-like protein (LONP)	U02389	1.7	2.2
PSF1	DNA replication complex GINS protein PSF1; KIAA0186 gene product	NM_021067	-1.6	-2.3
PSPH	phosphoserine phosphatase	NM_004577	1.6	1.8
PTPN12	protein tyrosine phosphatase, non-receptor type 12	NM_002835	1.5	1.7
RAB7L1	RAB7, member RAS oncogene family-like 1	BG338251	1.5	1.6
RABGGTB	Rab geranylgeranyltransferase, beta subunit	U49245	1.5	1.9
RAFTLIN	raft-linking protein	D42043	1.9	2.0
RAMP (ZNF198)	RA-regulated nuclear matrix-associated protein	AK001261	-2.1	-3.0
RAPH1	Ras association (RalGDS/AF-6) and pleckstrin homology domains 1, Lamellipodin (LPD)	AA194149	2.2	4.3
RASD1	RAS, dexamethasone-induced 1	AF069506	2.4	2.6
RASSF3	Ras association (RalGDS/AF-6) domain family 3	AI628605	1.5	2.0
RAVER1	RAVER1	BC002848	-1.5	-1.6
RB1CC1	RB1-inducible coiled-coil 1	BG402105	1.7	1.9
RBM6	RNA binding motif protein 6	AI041522	2.1	1.5
RBM8A	RNA binding motif protein 8A	AF182415	-1.7	-1.9
RCN1	reticulocalbin 1, EF-hand calcium binding domain	NM_002901	1.6	1.7
RDH11	retinol dehydrogenase 11 (all-trans and 9-cis)	AF167438	1.8	1.7
RECQL4	RecQ protein-like 4	NM_004260	-1.7	-1.7
RERG	RAS-like, estrogen-regulated, growth inhibitor	AW294092	1.8	1.5
RFC3	replication factor C (activator 1) 3, 38kDa	NM_002915	-1.7	-3.0
RHOB (ARHB)	ras homolog gene family, member B	AI263909	-1.6	-1.8
RHOBTB1	Rho-related BTB domain containing 1	AB018283	1.8	1.8
RHOBTB3	Rho-related BTB domain containing 3	BE620739	1.7	1.7
RHOQ	ras homolog gene family, member Q; ARHQ; RAS-LIKE PROTEIN TC10; TC10	BF670447	2.0	1.9
RKHD1	ring finger and KH domain containing 1	C18318	-2.2	-1.9
RNF159 (PCGF5)	ring finger protein (C3HC4 type) 159; polycomb group ring finger 5	AL045882	1.7	1.7
RNF19	ring finger protein 19 (Dorfin)	AB029316	1.6	1.5
RNF26	ring finger protein 26	AB055622	-1.6	-1.6
RNF6	ring finger protein (C3H2C3 type) 6	NM_005977	1.6	2.7
RREB1	ras responsive element binding protein 1	AW118862	1.7	1.6
RRM2	ribonucleotide reductase M2 polypeptide	BC001886	-1.6	-2.5
RTCD1	RNA terminal phosphate cyclase domain 1	NM_003729	1.9	2.1
RW1	RW1 protein	D87446	1.7	2.0
S100P	S100 calcium binding protein P	NM_005980	5.1	5.8
SAMD1 (LOC90378)	sterile alpha motif domain containing 1; atherin	BF207100	-1.7	-1.7
SARS	Seryl-tRNA synthetase; CDNA FLJ12277 fis, clone MAMMA1001711	AU147785	4.0	4.4
SAS	sarcoma amplified sequence	NM_005981	1.7	2.1
SAT	spermidine/spermine N1-acetyltransferase	BE326919	1.8	1.6
SC4MOL	sterol-C4-methyl oxidase-like	AV704962	2.4	2.5
SC5DL	sterol-C5-desaturase (ERG3 delta-5-desaturase homolog, fungal)-like	D85181	1.6	1.5
SCD	stearoyl-CoA desaturase (delta-9-desaturase)	AF116616	1.5	1.8
SEC24A	SEC24 related gene family, member A (S. cerevisiae)	BE645231	1.6	1.8
SEC24D	SEC24 related gene family, member D (S. cerevisiae)	NM_014822	1.8	1.7
SENP6	SUMO1/sentrin specific protease 6	AF306508	1.6	1.7
SESN2	sestrin 2	BF131886	2.8	2.4
SFMBT2	Scm-like with four mbt domains 2	T65020	2.0	2.5
SFN	stratifin	BC000329	-1.7	-1.6
SH3KBP1	SH3-domain kinase binding protein 1	AF230904	1.9	1.7
SHMT2	serine hydroxymethyltransferase 2 (mitochondrial)	NM_005412	1.7	2.4
SKP2	S-phase kinase-associated protein 2 (p45) (F-box protein Skp2; Cyclin A/CDK2-associated protein p45) (p45skp2)	BC001441	-1.6	-1.6
SLC16A14	solute carrier family 16 (monocarboxylic acid transporters), member 14	R15072	1.6	1.5
SLC1A4	solute carrier family 1 (glutamate/neutral amino acid transporter), member 4	AI889380	1.6	1.6
SLC29A1 (ENT1)	solute carrier family 29 (nucleoside transporters), member 1	AF079117	-1.9	-2.3
SLC2A10	solute carrier family 2 (facilitated glucose transporter), member 10	NM_030777	1.7	1.9
SLC33A1	solute carrier family 33 (acetyl-CoA transporter), member 1	BE464756	1.5	2.3
SLC35B3	solute carrier family 35, member B3	AL355815	1.6	1.6
SLC38A2	solute carrier family 38, member 2	NM_018976	2.0	2.0
SLC39A14	solute carrier family 39 (zinc transporter), member 14	D31887	1.6	1.6

Gene Symbol	Gene Title	Accession No.	Fold Change # 1	# 2
SLC3A2	solute carrier family 3 (activators of dibasic and neutral amino acid transport), member 2	NM_002394	2.1	2.4
SLC4A5	solute carrier family 4, sodium bicarbonate cotransporter, member 5	BG324504	1.6	1.9
SLC7A1	solute carrier family 7 (cationic amino acid transporter, y+ system), member 1	AA148507	1.8	1.7
SLC7A11	solute carrier family 7, (cationic amino acid transporter, y+ system) member 11	AA488687	5.7	6.2
SLC7A2	solute carrier family 7 (cationic amino acid transporter, y+ system), member 2	AA876372	1.7	1.9
SLC7A5	solute carrier family 7 (cationic amino acid transporter, y+ system), member 5	AB018009	2.0	2.3
SLITRK6	SLIT and NTRK-like family, member 6	AI680986	2.4	3.4
SNRPA1	small nuclear ribonucleoprotein polypeptide A'	AJ130972	-1.6	-1.8
SORL1 (C11orf32)	sortilin-related receptor, L(DLR class) A repeats-containing	AV728268	1.5	1.9
SOS1	son of sevenless homolog 1 (Drosophila)	AA700167	1.7	2.0
Spc24	kinetochore protein Spc24	AI469788	-1.8	-2.2
SPTLC2	serine palmitoyltransferase, long chain base subunit 2	U15555	1.6	2.0
STC2	stanniocalcin 2	BC000658	2.3	2.6
STCH	stress 70 protein chaperone, microsome-associated, 60kDa	NM_006948	2.7	2.3
STX3A	syntaxin 3A	BE966922	1.6	1.7
SUPT16H (FACT)	suppressor of Ty 16 homolog (S. cerevisiae)	AK024072	-1.7	-1.8
SYTL1	synaptotagmin-like 1	AI341537	1.9	1.8
TANC (KIAA1728 protein)	TPR domain, ankyrin-repeat and coiled-coil-containing; KIAA1728 protein	AB051515	1.8	1.8
TANK	TRAF family member-associated NFKB activator (ITRAF)	U59863	2.3	2.2
TBL1X (TBL1)	transducin (beta)-like 1X-linked	AW968555	1.9	1.8
TES	testis derived transcript (3 LIM domains)	NM_015641	1.7	1.9
TIGA1	TIGA1	BF314746	1.6	1.7
TK1	thymidine kinase 1, soluble	BC007986	-1.7	-2.8
TKT	transketolase (Wernicke-Korsakoff syndrome) (Glycoaldehyde transferase)	AU152969	1.6	1.6
TLE1	transducin-like enhancer of split 1 (E(sp1) homolog, Drosophila)	BE302305	1.8	1.7
TMC4	transmembrane channel-like 4	BE645551	1.7	1.6
TMEM41B (KIAA0033)	transmembrane protein 41B; KIAA0033 protein	N64760	1.5	1.5
TMEM64	transmembrane protein 64; hypothetical protein DKFZp762C1112	BG535396	-1.8	-2.6
TMPO	thymopoietin	AF113682	-1.6	-1.8
TncRNA	trophoblast-derived noncoding RNA	AU155361	1.6	2.5
TNFRSF10B	tumor necrosis factor receptor superfamily, member 10b	AF016266	2.7	2.5
TNRC9	trinucleotide repeat containing 9	AK025084	2.1	3.4
TOM1L1	target of myb1-like 1 (chicken)	NM_005486	2.0	1.7
TP53INP1	tumor protein p53 inducible nuclear protein 1	AW341649	3.1	3.5
TRGV9 (TARP)	T cell receptor gamma variable 9	M27331	-1.5	-1.7
TRIB1 (SKIP1; GIG2)	tribbles homolog 1 (Drosophila); G-protein-coupled receptor induced protein GIG2	NM_025195	1.6	1.7
TRIB3 (SKIP3, NIPK)	tribbles homolog 3 (Drosophila); neuronal cell death-inducible putative protein kinase	NM_021158	1.6	1.7
TRIP4	thyroid hormone receptor interactor 4	NM_016213	1.5	1.5
TROAP	trophinin associated protein (tastin)	NM_005480	-1.5	-1.6
TTC17	tetratricopeptide repeat domain 17	NM_018259	1.5	2.1
TTC3	tetratricopeptide repeat domain 3	D83077	1.8	2.1
TUBB (TUBB5, OK/SW-cl.56)	tubulin, beta polypeptide (beta5)	BC001002	-1.5	-2.6
TUBB2	tubulin, beta, 2	BC004188	-1.6	-1.9
TUBB3 (TUBB4)	tubulin, beta, 3 // tubulin, beta 4	AL565749	-1.6	-1.7
TXNIP	thioredoxin interacting protein	AA812232	2.0	1.5
TXNRD1	thioredoxin reductase 1	NM_003330	1.5	1.6
TYMS	thymidylate synthetase	AB077208	-2.4	-3.4
UBE2C	ubiquitin-conjugating enzyme E2C	NM_007019	-1.6	-2.0
UBLCP1 (MGC10067)	ubiquitin-like domain containing CTD phosphatase 1; hypothetical protein MGC10067	BF965546	1.5	1.5
UBR1	ubiquitin protein ligase E3 component n-recognin 1	AV715153	1.8	1.9
Ufm1	ubiquitin-fold modifier 1	NM_016617	1.6	1.6
UHRF1	ubiquitin-like, containing PHD and RING finger domains, 1	AK025578	-2.7	-2.7
UNC5B	unc-5 homolog B (C. elegans)	AK022859	2.1	3.3
UNQ1912	HGS_RE408	AI816071	1.8	1.6
USP3	ubiquitin specific protease 3; Ubiquitin carboxyl-terminal hydrolase 3 (EC 3.1.2.15)	AF077040	1.8	1.8
VAV3	vav 3 oncogene	NM_006113	1.6	2.0
VEGF	vascular endothelial growth factor	AF022375	4.2	4.6
WARS	tryptophanyl-tRNA synthetase	NM_004184	2.2	2.5
WDR72 (FLJ38736)	WD repeat domain 72	Z98443	1.7	1.7

Gene Symbol	Gene Title	Accession No.	Fold Change # 1	# 2
WIPI49 (FLJ10055)	hypothetical protein FLJ10055; WD40 repeat protein Interacting with phosphoInositides of 49kDa	AW052084	2.1	3.5
WSB1	WD repeat and SOCS box-containing 1	AL110243	1.6	1.7
XPOT	exportin, tRNA (nuclear export receptor for tRNAs)	AI984005	2.2	2.3
YARS	tyrosyl-tRNA synthetase	AW245400	1.7	2.0
YIPF5 (SMAP-5)	Yip1 domain family, member 5; golgi membrane protein SB140	AW473802	1.6	2.1
YPEL2	yippee-like 2 (Drosophila)	BE502982	2.2	3.7
YPEL5 (CGI-127)	yippee protein; yippee-like 5 (Drosophila)	NM_016061	1.6	1.7
YWHAH	tyrosine 3-monooxygenase/tryptophan 5-monooxygenase activation protein, eta polypeptide	NM_003405	-1.9	-1.8
ZFP36L2	zinc finger protein 36, C3H type-like 2	AI356398	-2.1	-3.1
ZIC4	Zic family member 4; Clone IMAGE:5301781, mRNA	AI367357	1.7	1.7
ZNF216	zinc finger protein 216	AF062347	1.5	1.6
ZNF277	zinc finger protein (C2H2 type) 277	NM_021994	1.6	1.8
ZNF367	zinc finger protein 367	N62196	-2.0	-2.8
ZWINT	ZW10 interactor	NM_007057	-1.7	-2.5
