# Supplementary material for: Gene expression profiling reveals effects of Cimicifuga racemosa (L.) NUTT. (black cohosh) on the estrogen receptor positive human breast cancer cell line MCF-7
Source: BMC Pharmacol. 2007 Sep 20;7:11. doi: 10.1186/1471-2210-7-11 (PMC2194763; doi:10.1186/1471-2210-7-11)
Supplement: Additional file 2 — Aryl hydrocarbon receptor activity. The diagram presents the results of a XRE-dependent reporter gene assay in rat H4IIE cells. [file 1471-2210-7-11-S2.pdf]

ADDITIONAL FILE 2

Gene expression profiling reveals effects of *Cimicifuga racemosa* (L.) NUTT. (black cohosh) on the estrogen receptor positive human breast cancer cell line MCF-7

Friedemann Gaube, Stefan Wölfl, Larissa Pusch, Torsten C Kroll and Matthias Hamburger

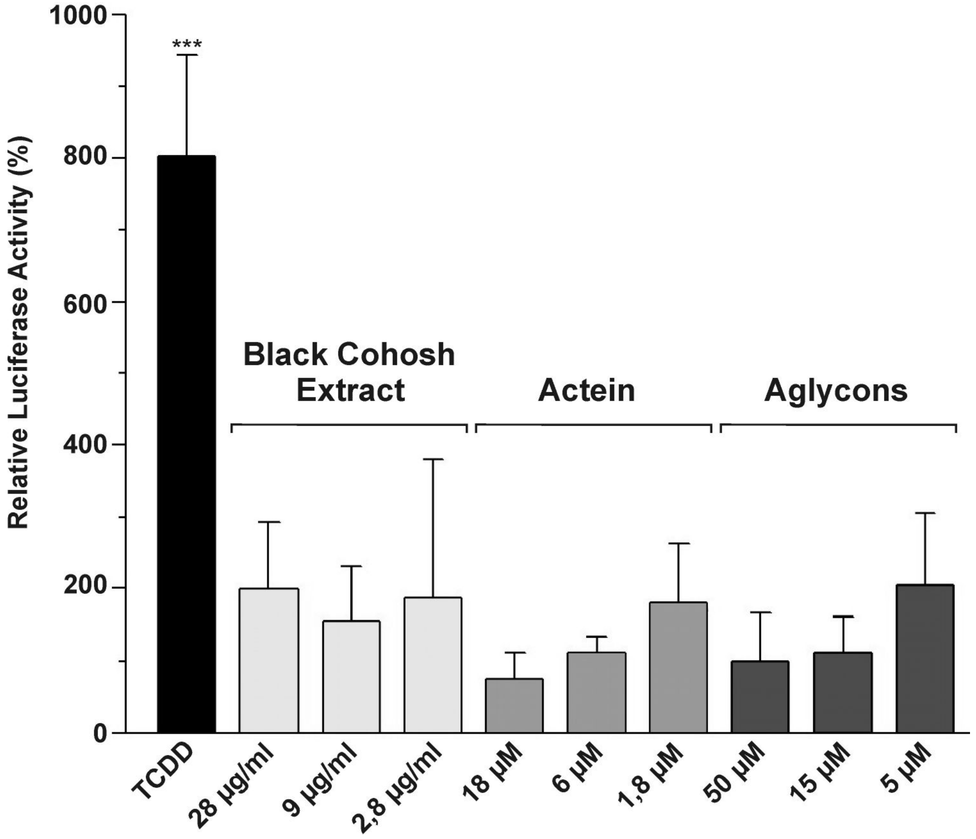

Rat hepatoma H4IIE cells, co-transfected with two xenobiotic responsive elements (XREs) and a luciferase gene (*P. pyralis*), were treated with different concentrations of black cohosh extract, actein, aglycons as well as solvent control (0,5 % DMSO) or 1 nM TCDD (2,3,7,8-tetrachlorodibenzo-*p*-dioxin) as positive control. Luciferase activity was measured spectrophotometrically. Relative luciferase activity values were calculated as percentage of solvent control (0.5 % DMSO value = 100 %). Data are presented as means ± SD (* $p < 0.001$ *vs*. DMSO control, Student´s t-test).

Except for TCDD no statistically significant alteration of luciferase activity *vs*. DMSO control was observed.
